# Novel bioinformatics approaches to design epitope-based vaccine against HIV latency by inquiring CTL epitopes and built-in adjuvants

**DOI:** 10.1038/s41598-025-17196-7

**Published:** 2025-09-25

**Authors:** Maryam Mashhadi Abolghasem Shirazi, Mina Hanan, Arash Arashkia, Seyed Mehdi Sadat

**Affiliations:** 1 Department of Molecular Virology, Pasteur Institute of Iran, Tehran, Iran; 2 Department of Hepatitis, AIDS and Blood borne Diseases, Pasteur Institute of Iran, No: 69, Pasteur Ave, Hepatitis, Tehran, Iran

**Keywords:** HIV-1, Latency, CTL epitopes, Gag p24 and pol and tat, Β-defensin, Bioinformatics tools, Molecular engineering, Infection

## Abstract

A key characteristic of HIV-1 persistence under antiretroviral therapy is its ability to form a latent reservoir in T cells, creating an obstacle to the virus eradication, therefore, there is no effective and permanent treatment against HIV-1. This study aims to develop an epitope-based vaccine that targets HIV-1’s key CTL epitopes involved in infection and latency. After studying and carefully checking previous reports and immunoinformatics, a multi-epitope vaccine was designed with strong CTL (Gag p24 and Pol) epitopes. CPP peptide (Tat) and an adjuvant (β-defensin) were incorporated into the vaccine construct for cell entry and to improve immunogenicity. The designed vaccine was evaluated in terms of antigenicity, allergenicity, safety, various physicochemical properties, solubility, and molecular docking with TLR4 using in silico tools. Immune simulation predicted a significant induction of immune responses. Successful expression and purification of the recombinant protein in *E. coli* BL21 strain, followed by confirmation by Western blot, demonstrated the feasibility of the vaccine production. Finally, our findings suggest that the designed epitope-based vaccine can induce humoral and cellular immune responses and is a suitable candidate for an HIV-1 therapeutic vaccine to control infection and latency.

## Introduction

Despite significant advancements in HIV-1 pathogenesis and virology, the lack of a prophylactic vaccine or curative treatment persists, affecting over 35 million people worldwide and resulting in approximately one million annual deaths. Furthermore, access to effective antiretroviral therapy remains limited, with only an average of 60% of infected individuals receiving adequate treatment^1,2^. While antiretroviral therapy (ART) is successful at controlling HIV-1, it requires ongoing treatment due to a hidden reservoir formed during the initial infection^3,4^. This reservoir mainly exists in memory CD4 + T cells and holds inactive provirus that avoids being detected by the immune system^5^. The challenge to finding a cure lies in HIV-1’s capacity to create latency in these enduring memory T cells, even when ART is working effectively^6^. The ability of these proviruses to become active again, start making copies, and generate infectious virus particles presents a major risk of viral rebound when ART is stopped. The reasons behind HIV-1 latency are complex and include factors like the low presence of important nuclear host transcription factors, such as NFκB and NFAT, in resting CD4 + T cells. There is also a lack of Tat and necessary cofactors for ongoing transcription, along with changes in gene regulation that inhibit viral gene expression^6,7^.

The innate immune system is pivotal in the initial defense against infections and the control of established HIV-1 infection. Consequently, research exploring innate immunity enhancement has become increasingly prominent in HIV-1 treatment strategies. Toll-like receptors (TLRs), are a family of pattern recognition receptors (PRRs) that are responsible for recognizing pathogen-associated molecular patterns (PAMPs)^8^, trigger interferon production, a primary antiviral response. Therefore, TLR agonists are considered promising candidates due to their potential to both reverse viral latency and modulate immune responses^9^. Currently, approved human vaccines containing TLR4, TLR7, and TLR9 agonists are available^10^. The adjuvant that has been used as a TLR4 agonist in approved human vaccines such as Gardasil is monophospholipid (MPL), which induces an appropriate immune response^11^. β-defensin peptides, functioning as TLR4 agonists, represent a class of antimicrobial peptides that contribute to host innate immunity. These peptides stimulate innate immune responses by recruiting naïve T cells and immature dendritic cells (DCs) through interactions with relevant immune receptors, including CCR6 and TLRs^12,13^. Remarkably, Kim et al.^14^ demonstrated that human β-defensin-2 can elicit primary antiviral innate immune responses and potentially mediate antigen-specific immunity. Furthermore, in silico studies employing immunoinformatics have successfully designed multi-epitope vaccines for Kaposi’s sarcoma, incorporating β-defensin as an N-terminal adjuvant to enhance immunogenicity^15^. Built-in adjuvants are used as immune response enhancers and suitable protein carriers that can effectively stimulate the immune system against the target pathogen by binding with the desired epitopes in an epitope vaccine^12^. The activation of immune cells at the same time, triggered by TLR agonists, can help remove infected cells and boost the host’s capability to manage HIV-1 infection. This process highlights how TLR agonists could be useful in creating effective treatments that enhance the immune system’s ability to control the virus^9^.

A new approach to re-transcribing proviruses involves utilizing CTL epitopes. This is important because strong cellular immune responses help manage the virus by reducing its levels in the body^16^. Early research is starting to look into how the specificity of CTL epitopes influences control over HIV-1. The goal is to find out if targeting specific viral proteins by CTLs can be a more effective way to reduce the virus^16,17^. CTL-based HIV-1 therapeutic vaccine significantly inhibits some Gag p24 epitopes such as TL9, KF11 and QW9 that interact with major HLAs such as HLA-A*02, HLA-A*03 and HLA-B*07, were non-mutable in the latent reservoir of chronic ART-treated subjects and suppressed viral growth when targeted by CTLs^18^. The observed data suggest that CTLs exhibit a higher affinity for Gag p24 epitopes relative to other viral proteins, including Env. This increased interaction between CTLs and Gag p24 is associated with superior viral replication control, potentially modulated by biophysical conditions^19^.

Pol is another region that contains CTL epitopes, but they are mostly present in the RT and IN regions, and this shows the importance of the presence of the Pol region in the design of a vaccine candidate^18^. Specific Pol epitopes, such as GKKAIGTVL (PR 68–76) and IAMESIVIW (RT 375–383), have been shown to elicit CTL responses^20^. These findings indicate the immunogenicity of all three Pol regions and suggest their potential role in viremia control and viral setpoint establishment during primary HIV-1 infection^17^. Studies have further identified conserved Pol epitopes, such as IETVEPVKL (RT 5–12) and SVPLDEGFRK (RT 117–126), as promising immunogens for HIV-1 vaccine candidates, demonstrating cross-protection^21^. These conserved epitopes elicit dominant immune responses, potentially offering protection against diverse HIV-1 subtypes^22^. Consistent with these observations, Pol epitopes have been shown to effectively stimulate CTL responses in various vaccine studies^23,24^.

The cell membrane’s hydrophobic characteristics primarily hinder the drug from entering the cell, which lessens its effectiveness. To overcome this, both natural and synthetic cell-penetrating peptides (CPPs) are commonly used as vehicles. They help deliver the drug into the cell through processes like clathrin-independent endocytosis or macropinocytosis, which aim to reduce the spread of pathogens while preserving the drug’s strong inhibitory effects^25^. Since viruses can only thrive inside host cells, many CPPs that originate from viruses serve as delivery vectors, help to penetrate the host cell. A notable example is HIV-1 Tat, which facilitates the transport of peptides that block ribosomal frameshifting, thereby decreasing the replication of pathogens^26^.

In this study, it is expected that the bioinformatics investigation will pave the way to obtain a suitable platform for the development of a long-term HIV-1 therapeutic vaccine as a way to enhance HIV-1 immune control. We have designed a multi-epitope construct containing three repeats of CTL epitopes, β-defenisin-2 as a built-in adjuvant, HIV-1 Tat as a CPP and evaluated all physiochemical parameters along with its immunogenicity and immune response simulation. Furthermore, we have investigated in silico the structural stability and interaction with the receptor through molecular dynamics (MD) and molecular docking simulations. Finally, we have successfully generated recombinant protein of HIV using the pET expression vector and *E. coli* (BL21) system.

## Materials and methods

### Retrieval of protein sequences and construction of a vaccine candidate

**Fig. 1:**
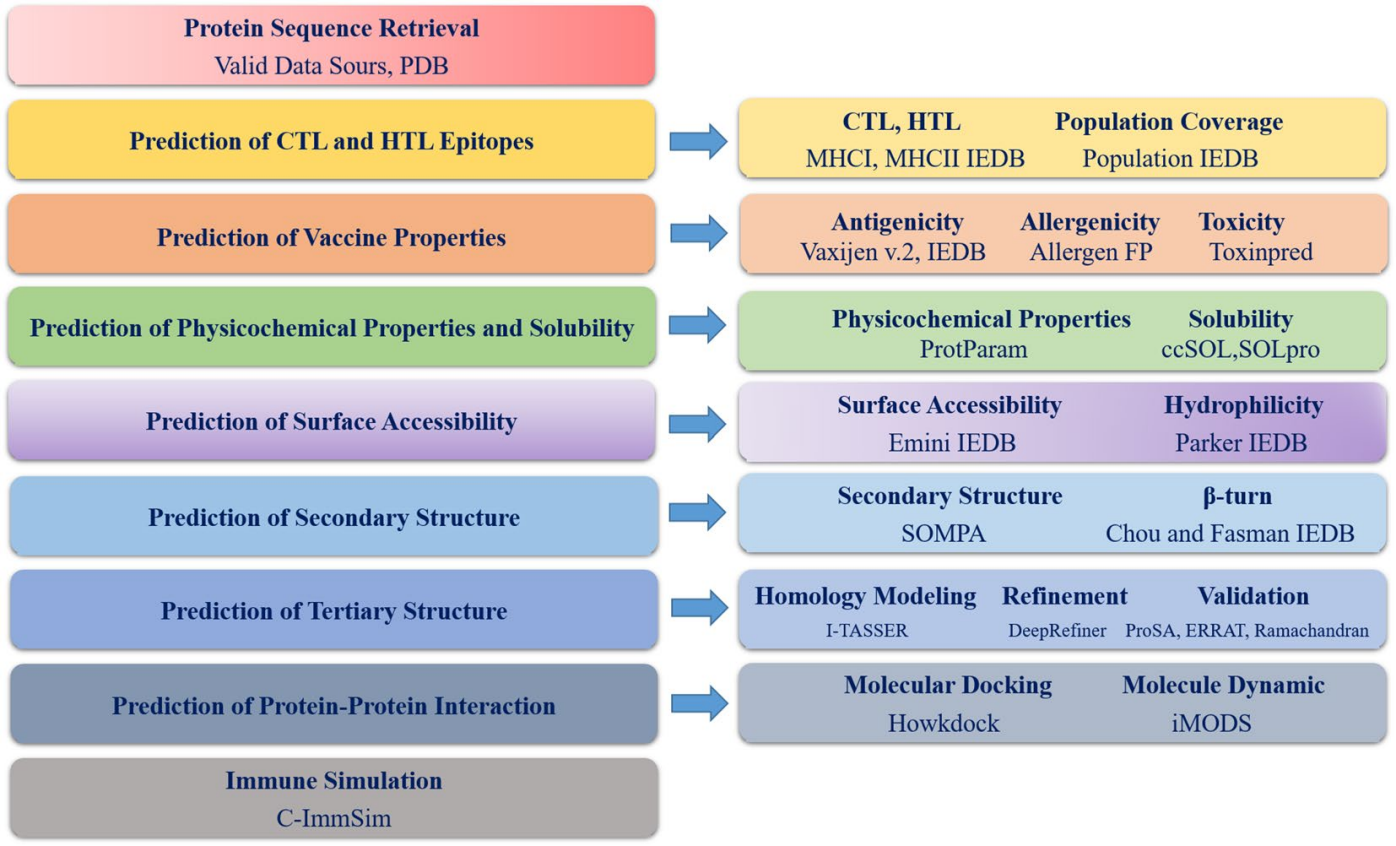
The flow chart of this study with tools used.

The flow chart of the performed procedures is shown in Fig. 1 in the current study. The previously reported sequences of Gag p24^19^, Pol^17^, Tat peptide (CPP)^27^ of HIV-1, β-defenisin-2 (the TLR4 agonist)^14^, 6xHis-tag^28^ and the rigid linkers (EAAAK)_4_, (AAY)^29^ were selected from valid data sources. Mouse TLR4 (PDB ID. 3VQ2) sequence, used for molecular docking analyses, was retrieved from the Protein Data Bank (PDB) database (http://www.rcsb.org)^30^. HLA-B*07:02 and HLA-A*25:01 were retrieved from the IPDIMGT/HLA (https://www.ebi.ac.uk/ipd/imgt/hla/) (31).

**Fig. 2:**
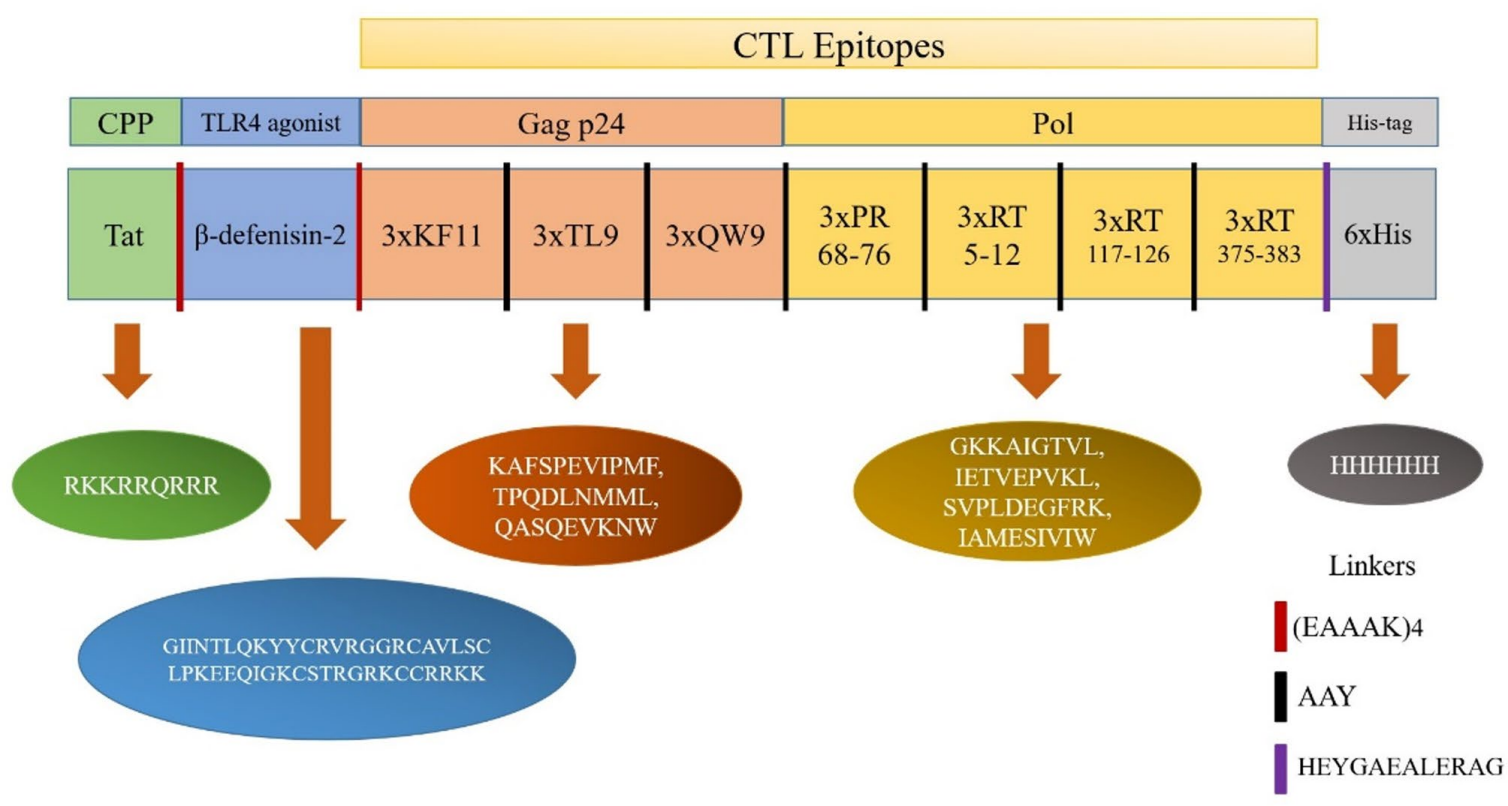
Schematic view of the platform. The Immunogenic platform contained the HIV-Tat (as a CPP) and β-defenisin-2 (the TLR4 agonist) tandem linked by short rigid linkers of (EAAAK)_4_ presented as the red dashes. Therefore, the 3x tandem repeats of the CTL epitopes Gag (KF11, TL9 and QW9) and Pol (PR; 68–76 aa, RT; 5–12 aa, 117–126 aa, 375–383 aa), tandem linked by short rigid linkers of AAY presented as the black dashes. The C-terminal was His-tag (6xHis) for facilitating purification of the antigen linked by HEYGAEALERAG linker are shown. The displayed sequences indicate the amino acid position of each fragment.

The vaccine construct, depicted in Fig. 2, was designed with an N-terminal methionine residue, followed by an HIV-Tat peptide (as the CPP) linked via an (EAAAK)_4_ linker. The inclusion of HIV-Tat, positioned upstream of the β-defensin-2 TLR4 agonist, aimed to enhance cell penetration and MHC class I processing^27^. Subsequently, β-defenisin-2 (the TLR4 agonist)^14^ linked to HIV-1 CTL epitopes by an AAY linker. Then, the three tandem copies of the epitopes Gag (KF11, TL9 and QW9) and Pol (PR; 68–76, RT; 5–12, 117–126, 375–383) were utilized to design the vaccine construct. Finally, a His-tag, facilitating purification, was appended to the C-terminus via an HEYGAEALERAG linker. These AAY and HEYGAEALERAG linkers were strategically included to facilitate proteasomal and lysosomal processing of the vaccine, respectively^27^.

### Prediction of cytotoxic and helper T lymphocytes epitope

CTL epitope prediction is crucial for subunit vaccine development. The amino acid sequence was evaluated using the IEDB MHC-I (http://tools.iedb.org/mhci/) tool for the prediction the CTL epitopes. The HLA-A such as HLA-A*25:01, and HLA-B such as HLA-B*58:01, HLA-B*57:01, HLA-B*35:01, HLA-B*07:02, HLA-B*52:01 human alleles^17,19^. H-2Db, H-2Dd, H-2Kb, H-2Kd, H-2Kk, and H-2Ld mouse alleles were selected to predict peptide binding to MHC-I. Epitope lengths were set as 9-mer separately for humans and mice^32^. A percentile rank ˃ 1 was acceptable.

The IEDB MHC-II (http://tools.iedb.org/mhcii/) server was employed for predicting Helper T lymphocytes (HTL) epitopes. The alleles were selected from human HLA-DR and HLA-DQ alleles, and mouse H-2IAb, H-2IAd, H-2IEd, H-2IEk, and H-2IEs. Epitope lengths were set as 15-mer separately for humans and mice^33^. A percentile rank ˃ 1 was acceptable.

### Prediction of discontinuous b‑cell epitope

Prediction of discontinuous B-cell epitopes was conducted in the final platform construct. Conformational epitopes are essential in the ligand-receptor interaction. To forecast these conformational B-cell epitopes, the Ellipro server located at (http://tools.iedb.org/ellipro/*)* was utilized, based on the confirmed 3D structure. This innovative web tool assesses epitopes based on the geometrical characteristics of the protein structure and provides a Protrusion Index (PI) score for each predicted epitope averaged across its residue locations. In the Ellipro approach, the 3D protein shape is modeled using multiple ellipsoids. When ellipsoids have a PI of 0.9, it indicates that approximately 90% of the protein residues are contained within, while around 10% are external to the ellipsoid^34^.

### Prediction of population coverage

The IEDB server (http://tools.iedb.org/population*)* was used to check how well vaccine candidate peptides fit with MHC class I and class II. This analysis aimed to measure their effectiveness in both the Iranian population and worldwide^35^.

### Prediction of antigenicity, allergenicity and toxicity of the platform

Determining the antigenic properties is one of the important processes during vaccine development. VaxiJen v2.0 (http://www.ddg-pharmfac.net/vaxijen/VaxiJen/VaxiJen.html) server was used to evaluate the antigenicity of the platform. The VaxiJen v2.0 algorithm identifies the protein as an antigen by analyzing its physicochemical properties and the threshold was 0.4^36^. The Kolaskar and Tongaonkar antigenicity (http://tools.iedb.org/bcell/result/) server was employed to predict the antigenicity of the epitope sequence^37^. Another determining factor in vaccine development is the identification of its allergenecity. AllergenFP (http://ddgpharmfac.net/AllergenFP/) server evaluated the allergenic characteristics of the vaccine construct. This server detects non-allergens and allergens based on protein sequence characteristics such as size, relative abundance, hydrophobicity, α-helix and the tendency to form beta chains^38^. The ToxinPred server, created by the Raghava group, was utilized to evaluate the toxicity of individual CTL epitopes and the complete vaccine design (http://crdd.osdd.net/raghava/toxinpred) server. This tool employs a support vector machine (SVM) classification model that has been trained on both toxic and non-toxic peptides that have been confirmed through experiments. For the toxicity predictions, we applied the “SVM-based method (Quantitative Matrix and Motif-based model)” while using the peptide toxicity prediction module. The sequences provided were processed with the default settings, allowing the server to determine the toxicity of the peptides based on their physicochemical characteristics, composition, and the presence of specific motifs^39^.

Thus, each epitope was analyzed independently for its antigenicity using the VaxiJen v2.0 server, toxicity with ToxinPred. Allergenicity with AllerCatPro 2.0 (https://allercatpro.bii.a-star.edu.sg/). This tool evaluates if a protein could be allergenic by checking its amino acid sequence and three-dimensional (3D) structure against known allergens from extensive databases like AllergenOnline, UniProt, and AllergenDB. Utilizing a mixed approach, the server such as screening for sequence similarities with known allergens using FASTA-based alignment andc omparing 3D structures, when data is accessible, to identify possible allergenic folds or epitopes. It employs a scoring system based on the weight of evidence, which looks at several factors (such as linear epitope similarity, matches of structural motifs, and protein family classification) to classify the input protein as: No evidence for allergenicity, Weak evidence, or Strong evidence for allergenicity^40^.

### Prediction of disulfide engineering

To forecast the disulfide bonds and Cys-Cys interactions that are crucial for stabilizing folding^41^, the DiANNA server (http://clavius.bc.edu/~clotelab/DiANNA/) was utilized.

To enhance the stability of the vaccine’s structure, modifications involving disulfide bonds were carried out using the Disulfide by Design 2 (DbD2) server (http://cptweb.cpt.wayne.edu/DbD2/*).* The improved 3D structure of the vaccine was submitted in PDB format for analysis, allowing for the identification of appropriate cysteine residue pairs that could create disulfide bridges. Criteria for these predictions focused on geometric requirements, which included a Chi3 dihedral angle ranging from − 87° to + 97° and Cβ–Cβ distances of 4.4 Å to 6.8 Å, adhering to standard guidelines for robust disulfide bond creation. Pairs with energy scores below 2 kcal/mol were deemed most effective in strengthening the vaccine’s structural integrity without interfering with the immune response areas^42^.

### Prediction of physicochemical properties and solubility of the platform

The ProtParam tool on the Expasy server (http://web.expasy.org/protparam/)^43^ was used to analyze the physicochemical characteristics of the chosen protein. This analysis included calculating various protein attributes like amino acid composition, molecular weight, aliphatic index, instability index, grand average of hydropathicity (GRAVY), and theoretical isoelectric point (pI). Additionally, to assess the solubility of highly expressed proteins in E. coli and to differentiate between soluble and insoluble proteins based on their physicochemical traits, the sequence was examined using the ccSOL (http://www.tartaglialab.com/)^44^ and SOLpro (http://scratch.proteomics.ics.uci.edu/)^45^ platforms.

###  Surface accessible regions and hydrophilicity prediction

CPPs (Cell Penetrating Peptides) are widely used as carrier molecules to deliver drugs into cells, so they must be located on the surface^26^. We analyzed the surface accessible regions and hydrophilicity of the epitope sequence prediction by using Emini surface accessibility prediction^46^ and Parker hydrophilicity prediction^47^ of IEDB (http://tools.iedb.org/bcell/result/) tools, respectively.

### Secondary structure prediction

The secondary structure of the platform was analyzed by the SOPMA (Self-Optimized Prediction Method with Alignment) (http://npsa-pbil.ibcp.fr) server. This tool is an online server for predicting coiled-coil regions, random coil regions, bend regions, globular regions, transmembrane helices, and solvent accessibility of the protein^48^. Several studies showed that the antigenic parts were positioned in beta-turn regions of a protein^49^. For the analysis of beta-turn regions Chou and Fasman prediction was used^50^.

### Tertiary structure and refinement prediction

The tertiary (3D) structure of the platform was generated using the I-TASSER (Iterative Threading ASSEmbly Refinement) server (http://zhanglab.ccmb.med.umich.edu/ITASSER/)^51^. The reliability of the predicted models was assessed using the C-score, a metric ranging from − 5 to 2, where higher values indicate increased confidence. To enhance the quality of the I-TASSER-derived model, the DeepRefiner server (http://watson.cse.eng.auburn.edu/DeepRefiner/)^52^ was employed for high-precision structure refinement using deep network calibration. The final refined model was visualized using PyMOL viewer.

### Validation of tertiary structure

Verifying the tertiary structure is a crucial part of the model-building process, as it helps to find potential mistakes in the predicted 3D models. For the analysis of these 3D models, three online servers were utilized. First, ProSA (https://prosa.services.came.sbg.ac.at/prosa.php*)* calculates a total quality score known as the Z-score for the given input structure. If the Z-score falls outside the accepted range typically seen in native proteins, it suggests that there might be errors in the structure (53). Second, the Ramachandran plot was generated using the Ramachandran plot server (https://zlab.umassmed.edu/bu/rama/index.pl*)*, which shows the quality of the 3D model by indicating the percentage of values within allowed and favored regions^54^. Lastly, ERRAT (http://services.mbi.ucla.edu/ERRAT/*)* was employed to predict non-bonded interactions among different types of atoms^55^.

### Molecular docking of the platform with immune receptor

Molecular docking simulations were performed to investigate the interaction between the antigenic molecule and the TLR4/MD2 co-receptor (PDB ID: 3VQ2, with LPS substitution), a key component in generating effective immune responses. The TLR4/MD2 structure was retrieved from the Protein Data Bank (PDB) (https://www.rcsb.org*).* Docking was carried out using the HawkDock server (http://cadd.zju.edu.cn/hawkdock/), where lower affinity scores indicate stronger interactions^56^. To provide a comparative baseline, the interaction between unincorporated β-defensin-2 and TLR4/MD2 (PDB ID: 3VQ2, with LPS substitution) was also assessed using the same docking protocol.

High affinity CTL epitopes were selected based on IEDB binding predictions and antigenicity. Then, peptide structures of CTL epitopes, HLA-B*07:02 and HLA-A*25:01 were modeled using PEP-FOLD3 (https://bioserv.rpbs.univ-paris-diderot.fr/services/PEP-FOLD3/*)*^57^. The structure of HLA-B*57:01 (PDB ID: 2BVP) was retrieved from the Protein Data Bank. Docking was carried out using the HawkDock server, where lower affinity scores indicate stronger interactions. The interaction between KF11 _ HLA-B 57:01 (PDB ID: 2BVP, with HIV substitution) and TL9 _ HLA-B 07:02 and Pol RT 5–12 _ HLA-A 25:01 was also assessed using the same docking protocol. Finally, Dimplot in LIGPLOT software was used for the analysis. This tool generates a plot that displays the patterns of hydrogen bonding and hydrophobic interactions between the ligand and the receptor^58^.

### Molecular dynamic simulation

A molecular dynamics simulation study was performed on the platform structure, selected based on its superior molecular docking performance. The iMODS server (http://imods.Chaconlab.org/), a freely available resource designed for rapid and accessible protein flexibility analysis, was employed for this simulation^59^.

#### Immune simulation against the platform

Agent-based simulations were conducted using the C-ImmSim server (http://150.146.2.1/C- IMMSIM/index. Php)^60^ to anticipate how the vaccine candidate interacts with the human immune system and to illustrate the subsequent immune response. This simulation focused on a vaccine schedule for HIV that included three shots given every four weeks. The parameters for the simulation were kept at their default settings, with the injections taking place on days 0, 28, and 35.

### Expression and purification of the Recombinant protein

The platform’s DNA sequence was synthesized in cloning/expression vector pET-28a (+), in *BamHI* and *EcoRI* restriction sites (Biomatik, Canada), and finally was transformed in *E. coli* BL21 (DE3) strain (Novagen, USA). Protein expression was induced with 1 mM IPTG (Sigma, UK) at 37 °C, and the cell pellet was harvested after 4 h. Successful protein expression was verified by SDS-PAGE and Western blot, employing an anti-6X His tag antibody (Abcam, UK) according to established methods^61^. The recombinant protein (RP) was purified under native conditions using a Ni-NTA agarose column (Qiagen, Germany), as per the manufacturer’s instructions^62^. Protein concentration was quantified using the Bradford method^63^ and endotoxin levels were determined using the chromogenic Limulus amoebocyte lysate (LAL) assay (BioWhittaker, UK), as per the manufacturer’s instructions.

## Results

### Prediction of CTL and HTL epitope

For the selected fusion protein, CTL (9-mer) epitopes were analyzed using by IEDB tool to predict the potential epitopes of the construct at the threshold value based on percentile rank. The results showed that CTL epitopes were able to bind to MHC-I with high antigenicity score, as obtained through analysis by Kolaskar and Tongaonkar tool. Human alleles predicted epitopes are HLA-B such as HLA-B*58:01, HLA-B*57:01, HLA-B*35:01, HLA-B*07:02, HLA-B*52:01 and HLA-A such as HLA-A*25:01. The CTL epitopes 17 peptides in mice (Table 1) and 26 peptides in human (Table 2) were predicted to binding to MHC-I molecules.

**Table 1:** Mice MHC-I binding epitope prediction.

No.	Protein	Peptide sequence	Allele	Start	End	Percentile score	Percentile rank	Antigenicity
1				269	277	0.748176	0.17	+
2	Pol (RT)	IETVEPVKL	H-2-Kk	260	268	0.748176	0.17	+
3				251	259	0.748176	0.17	+
4				170	178	0.672466	0.03	-
5	Gag (TL9)	TPQDLNMML	H-2-Ld	161	169	0.672466	0.03	-
6				152	160	0.672466	0.03	-
7	Pol (RT)	EPVKLIETV		264	272	0.38723	0.74	+
8				255	263	0.38723	0.74	+
9				170	178	0.382951	0.75	-
10	Gag (TL9)	TPQDLNMML	H-2-Kk	161	169	0.382951	0.75	-
11				152	160	0.382951	0.75	-
12				140	148	0.339561	0.02	-
13	Gag (KF11)	FSPEVIPMF	H-2-Dd	129	137	0.339561	0.02	+
14				118	126	0.339561	0.02	+
15				140	148	0.309749	0.28	+
16	Gag (KF11)	FSPEVIPMF	H-2-Kb	129	137	0.309749	0.28	+
17				118	126	0.309749	0.28	+

**Table 2:** Human MHC-I binding epitope prediction.

No.	Protein	Peptide sequence	Allele	Start	End	Percentile score	Percentile rank	Antigenicity
1				332	340			+
2			HLA-B*58:01	323	331	0.991063	0.01	+
3	Pol (RT)	IAMESIVIW		314	322			+
4				332	340			+
5			HLA-B*57:01	323	331	0.990786	0.01	+
6				314	322			+
7				200	208			-
8			HLA-B*57:01	191	199	0.952272	0.06	-
9	Gag(QW9)	QASQEVKNW		182	190			+
10				200	208			-
11			HLA-B*58:01	191	199	0.944439	0.04	-
12				182	190			+
13				170	178			-
14	Gag(TL9)	TPQDLNMML	HLA-B*07:02	161	169	0.708988	0.12	-
15				152	160			-
16	Pol (RT)	ETVEPVKLI	HLA-A*25:01	261	269	0.584606	0.06	+
17				252	260			+
18				170	178			-
19	Gag(TL9)	TPQDLNMML	HLA-B*35:01	161	169	0.576357	0.18	-
20				152	160			-
21				200	208			-
22	Gag(QW9)	QASQEVKNW	HLA-A*25:01	191	199	0.283418	0.25	-
23				182	190			+
24				332	340			+
25	Pol (RT)	IAMESIVIW	HLA-B*52:01	323	331	0.246657	0.23	+
26				314	322			+

High-binding MHC-II epitopes (15-mer) for mice and human alleles, predicted with the IEDB tool were determined as HTL epitopes based on percentile rank. Human alleles predicted epitopes are HLA-DR. The results showed that Pol (RT) and Gag p24 (KF11) CTL epitopes could act as a suitable T cell epitope that was capable of binding to different alleles of mice MHC-II (Table 3) and human (Table 4).

**Table 3:** Mice MHC-II binding epitope prediction.

No.	Protein	Peptide sequence	Allele	Start	End	Percentile rank	Antigenicity
1	Pol (RT)	KAAYIAMESIVIWIA	H-2-IAd	310	324	0.68	+
2	Pol (RT)	AAYIAMESIVIWIAM	H-2-IAd	311	325	0.95	+

**Table 4:** Human MHC-II binding epitope prediction.

No.	Protein	Peptide sequence	Allele	Start	End	Percentile rank	Antigenicity
1			HLA-DRB1*04:02	314	328	0.02	+
2				323	337		+
3	Pol (RT)	IAMESIVIWIAMESI	HLA-DRB1*11:02	314	328	0.25	+
4				323	337		+
5			HLA-DRB1*13:01	314	328	0.43	+
6				323	337		+
7	Gag(KF11)	KAFSPEVIPMFKAFS	HLA-DRB1*11:02	116	130	0.98	+
8				127	141		+

### Prediction of discontinuous (Conformational) b‑cell epitopes

**Fig. 3:**
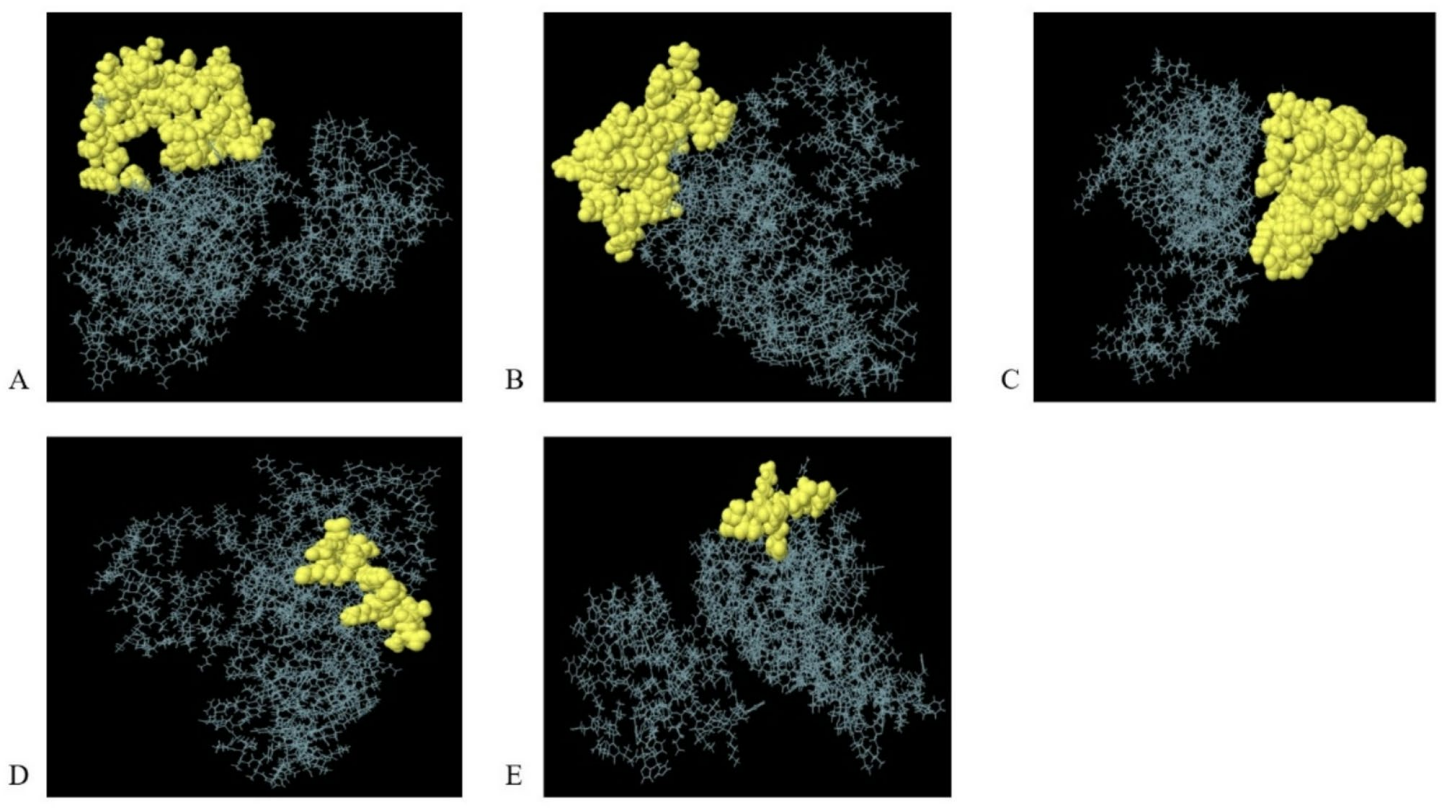
3D demonstration of the predicted conformational or discontinuous B cell epitopes in the platform. A yellow surface indicates the discontinuous B cell epitopes, and the rest of the residues are illustrated in grey sticks.

In the 3D model of the platform, residues with a value of 0.6 or higher were identified as conformational epitopes (Table 6). Also, discontinuous epitopes predicted in the 3D structure of the platform are shown (Fig. 3).

**Table 5:** Predicted discontinuous epitopes of the platform.

No.	Number of residue	Residue	Score
1	47	A: K58, A: Y59, A: Y60, A: C61, A: V63, A: R64, A: G65, A: G66, A: R67, A: C68, A: A69, A: V70, A: L71, A: S72, A: C73, A: L74, A: P75, A: K76, A: E77, A: E78, A: Q79, A: I80, A: G81, A: K82, A: C83, A: S84, A: T85, A: R86, A: G87, A: R88, A: K89, A: C90, A: C91, A: R92, A: K94, A: K95, A: E96, A: A97, A: A98, A: A99, A: K100, A: E101, A: A102, A: A103, A: A104, A: K105, A: A107	0.745
2	47	A: A114, A: K115, A: K116, A: A117, A: F118, A: S119, A: P120, A: E121, A: V122, A: I123, A: P124, A: M125, A: K127, A: Q200, A: A201, A: S202, A: Q203, A: E204, A: V205, A: K206, A: N207, A: W208, A: H209, A: E210, A: Y211, A: G212, A: A213, A: E214, A: A215, A: L216, A: E217, A: R218, A: A219, A: G220, A: G221, A: K222, A: K223, A: K231, A: K232, A: G235, A: T236, A: L238, A: G239, A: K240, A: K241, A: A242, A: I243	0.692
3	74	A: V266, A: K267, A: I269, A: E270, A: T271, A: V272, A: E273, A: P274, A: V275, A: K276, A: L277, A: A278, A: A279, A: Y280, A: S281, A: V282, A: P283, A: L284, A: D285, A: E286, A: G287, A: F288, A: R289, A: K290, A: S291, A: V292, A: P293, A: D295, A: G307, A: K310, A: A311, A: I314, A: A315, A: E317, A: S318, A: I319, A: V320, A: I321, A: W322, A: I323, A: A324, A: M325, A: E326, A: S327, A: I328, A: I330, A: W331, A: I332, A: A333, A: M334, A: E335, A: S336, A: I337, A: V338, A: I339, A: W340, A: H341, A: E342, A: Y343, A: G344, A: A345, A: E346, A: A347, A: L348, A: E349, A: R350, A: A351, A: G352, A: H353, A: H354, A: H355, A: H356, A: H357, A: H358	0.692
4	16	A: M1, A: E2, A: A3, A: A4, A: A5, A: K6, A: E7, A: A8, A: K11, A: A14, A: A15, A: K16, A: E17, A: A19, A: A20, A: K23	0.641
5	10	A: Q27, A: E31, A: A32, A: A33, A: A34, A: K35, A: A37, A: A38, A: A39, A: A180	0.634

**Table 6:** Population coverage based on combined MHC data.

No.	Population	Coverage^a^	Average Hit^b^	PC90^c^
1	Europe	81.87%	1.26	0.55
2	Iran	78.51%	1.2	0.47
3	North America	73.57%	1.05	0.37
4	West Indies	71.29%	1.05	0.35
5	Northeast Asia	68.64%	0.92	0.32
6	Oceania	64.95%	0.87	0.29
7	Southeast Asia	64.64%	0.87	0.28
8	East Africa	64.01%	0.89	0.28
9	South Asia	62.05%	0.85	0.26
10	Southwest Asia	61.06%	0.81	0.26
11	East Asia	57.07%	0.75	0.23
12	North Africa	55.76%	0.72	0.23

a) Projected population coverage.

b) Averaged number of epitope Hits/HLA combinations recognized by the population.

c) Minimum number of epitope Hits/HLA combinations recognized by 90% of the population.

### Prediction of population coverage

**Fig. 4:**
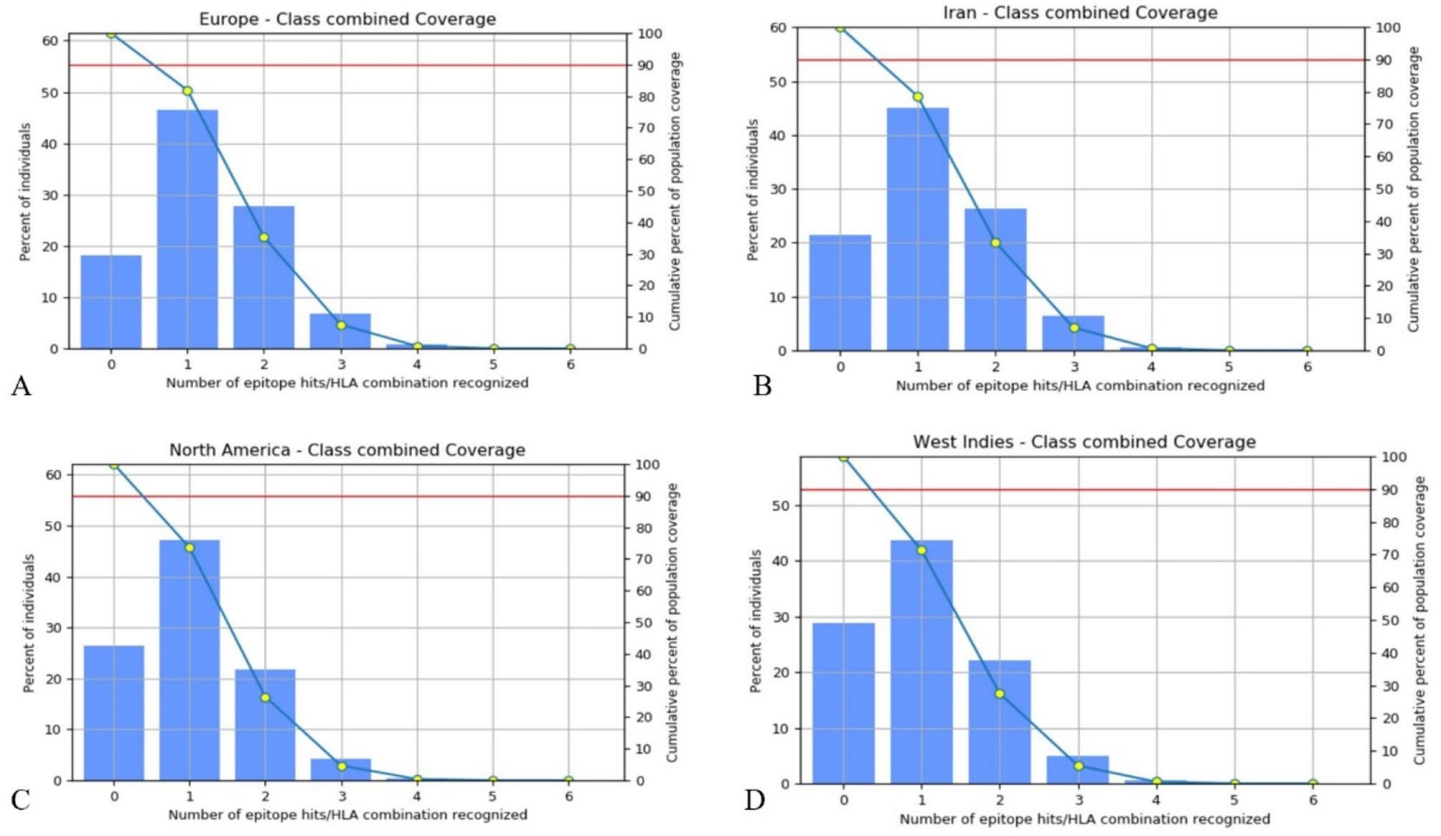
Population coverage based on MHC combined data. Different HIV-1 affected regions were selected for assessment of the population coverage of the platform CTL epitopes. In the graphs, the line (-o- as shown yellow color) show the cumulative percentage of population coverage of the epitopes; the bars show the population coverage for platform epitope. PC90 as shown red line is 90% population coverage.

Population coverage prediction, using identified MHC-I and MHC-II binders, was performed to estimate epitope responsiveness across diverse regions, with a focus on maximizing coverage of HIV-1 latently infected individuals, particularly within the Persian population. The population coverage analyses showed the highest 81.87% population coverage of the epitopes in Europe. The highest population coverage epitopes after Europe were 78.51% coverage in Iran, 73.57% and 71.29% coverage in North America and the West Indies, respectively (Fig. 4). The results of total population coverage are listed in Table 7.

**Table 7:** Antigenicity, allergenicity and safety of the platform predicated by bioinformatic analyses.

No.	Evaluated immunogenicity	Results
1	Antigenicity (Vaxijen v.2)	0.4153
2	Allergenicity (Allergen FP)	0.83 non-allergen
3	Toxicity (Toxinpred)	Non-toxic

### Prediction of antigenicity, allergenicity, and safety of the platform

**Fig. 5:**
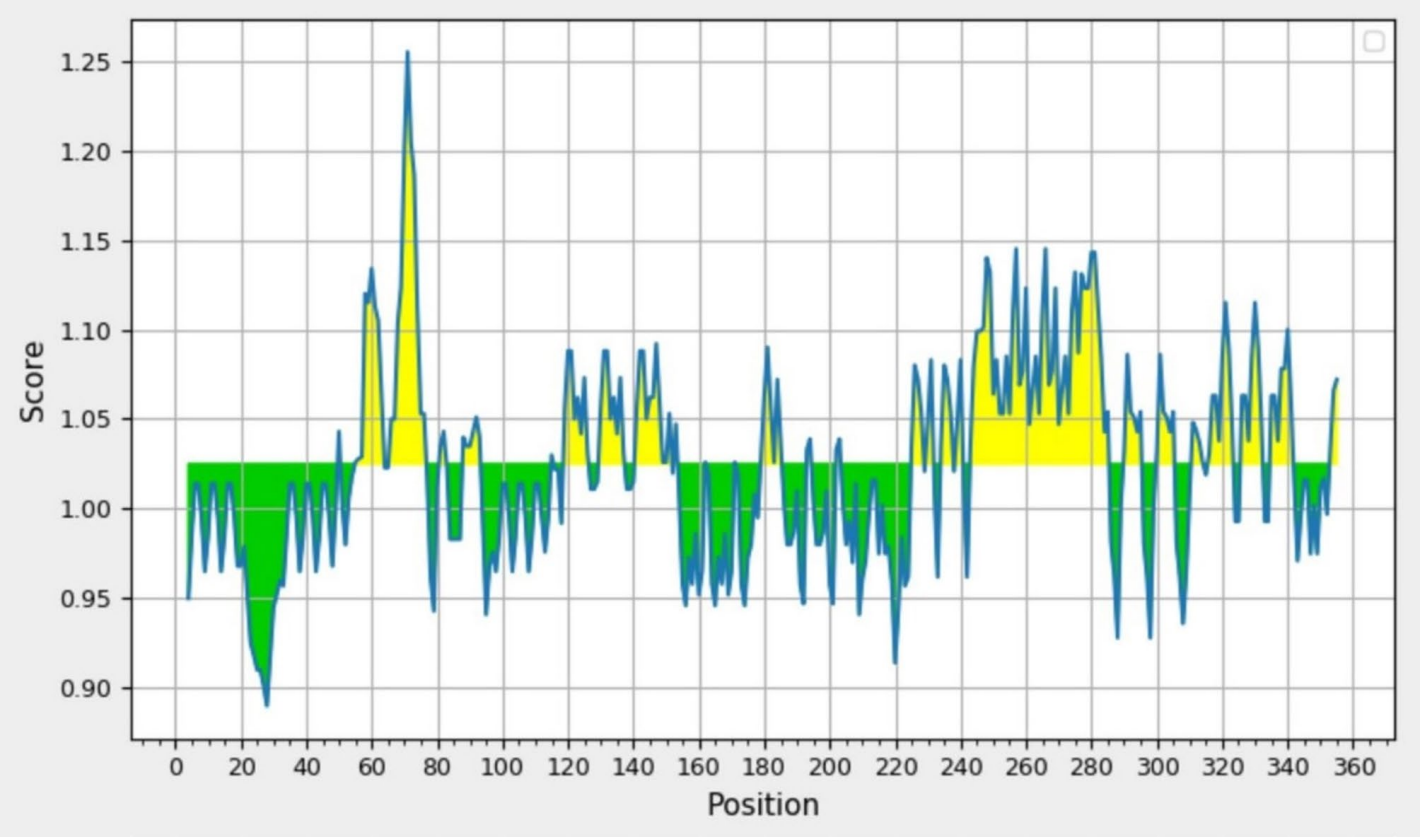
Antigenicity prediction by Kolashkar and Tongaonkar IEDB tool. The x-axis show the amino acid sequence position and y-axis show the antigenicity score. The threshold was 1.025. The yellow regions above the threshold are antigenic sequences, which are CTL epitope.

The platform was investigated for antigenicity by the VaxiJen v2.0 and Kolaskar and Tongaonkar tools, and it was found that the protein could be a good antigen. Utilizing the default threshold of 0.4 in VaxiJen v2.0, the platform demonstrated an antigenicity score of 0.4153 (Table 8), suggesting favorable antigenic properties. The platform sequence predicted on the Kolaskar and Tongaonkar tool showed the CTL epitopes were a good antigen (Fig. 5). The allergenic threshold above 0.759 is considered non-allergenic. The allergenicity score of the platform was 0.83, indicating it might be non-allergenic. The platform was checked for toxicity and found to be non-toxic (Table 8).

The antigenicity of each epitope was evaluated individually using the VaxiJen v2.0 server, while allergenicity was assessed through AllergenFP, and toxicity was analyzed with ToxinPred. By applying the standard threshold of 0.4 in VaxiJen v2.0, the scores for the antigen’s CTL epitopes—KF11, TL9, and Pol (RT) 5–12—along with β-defensin were recorded as 0.5106, 1.0473, 0.8406, and 0.6563, respectively. On the other hand, the non-antigen’s CTL epitopes QW9, Pol (PR) 68–76, Pol (RT) 375–383, and TAT (considered a CPP) achieved scores of 0.3400, −0.1639, 0.2360, and − 0.5547, respectively. The CTL and Tat epitopes were checked for allergenicity and toxicity and found to be no evidence for allergenicity and non-toxic (Table 9).

**Table 8:** Antigenicity, allergenicity and safety of the CTL and CPP epitopes predicated by bioinformatic analyses.

No.	Epitopes	Sequence	Antigenicity (Vaxijen v.2)	Allergenicity (Allergen FP)	Toxicity (Toxinpred)
1	Gag p24 (KF11)	KAFSPEVIPMF	Antigen	Non-allergen	Non-toxin
2	Gag p24 (TL9)	TPQDLNMML	Antigen	Non-allergen	Non-toxin
3	Gag p24 (QW9)	QASQEVKNW	Non-antigen	Non-allergen	Non-toxin
4	Pol (PR) 68–76	GKKAIGTVL	Non-antigen	Non-allergen	Non-toxin
5	Pol (RT) 5–12	IETVEPVKL	Antigen	Non-allergen	Non-toxin
6	Pol (RT) 375–383	IAMESIVIW	Non-antigen	Non-allergen	Non-toxin
7	Tat (CPP)	RKKRRQRRR	Non-antigen	Non-allergen	Non-toxin

**Table 9:** Physicochemical and solubility characteristics of the platform predicated by bioinformatic analyses.

No.	Evaluated characteristics	Results
1	No. of amino acids	358 aa
2	Molecular weight	39416.90 kDa
3	Instability index	38.41
4	Gravy	−0.266
5	Aliphatic index	81.45
6	Theoretical pI	9.25
7	Total No. of positively charged residues (Arg + Lys)	56
8	Total No. of negatively charged residues (Asp + Glu)	44
9	Solubility (SOLpro)	0.906038 Soluble
10	Solubility (ccSOL)	94% Soluble

### Prediction of physicochemical property and solubility

**Fig. 6:**
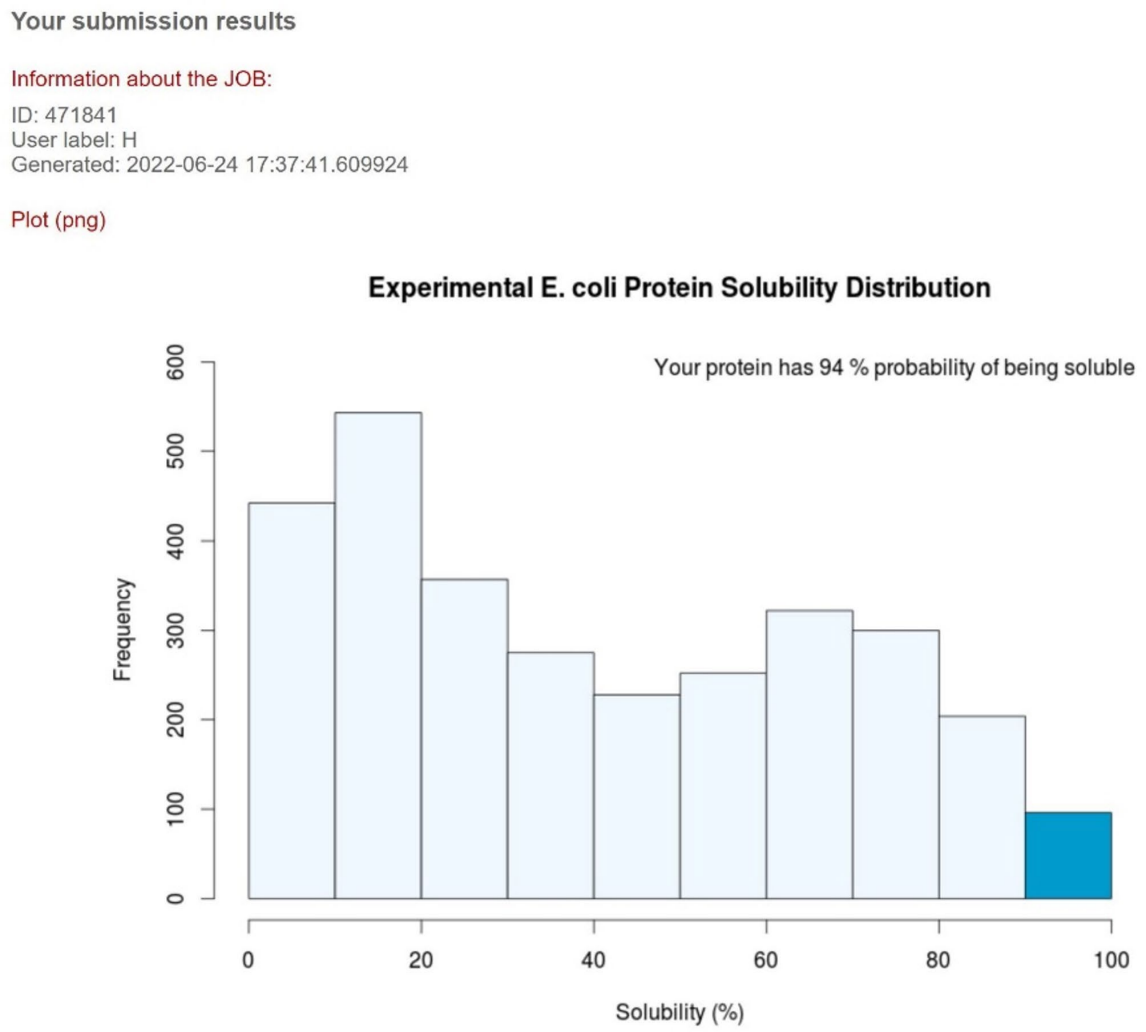
Experimental *E.coli* protein solubility distribution. The ccSOL server predicted the solubility of the protein based on physicochemical properties of amino acid sequence. The result of this server the protein was 94% soluble.

As detailed in Table 10, the platform construct exhibited a molecular weight of 39416.90 kDa and consisted of 358 amino acids. An instability index of 38.41, below the threshold of 40, indicated protein stability. The grand average of hydropathicity (GRAVY) was − 0.266, suggesting a hydrophilic nature. The aliphatic index was 81.45, and the theoretical isoelectric point (pI) was 9.25, with 56 positively charged and 44 negatively charged residues. Solubility predictions using SOLpro and ccSOL servers yielded scores of 0.906038 and 94%, respectively (Fig. 6), indicating high solubility in E. coli. Collectively, these bioinformatic analyses predicted the platform to be polar, hydrophilic, positively charged, stable, and soluble in *E. coli*.

**Table 10:** DiANNA-predicted disulfide bonds epitopes.

No.	Cysteine sequence	Distance	Bond	Score
1	73–83	10	CAVLSCLPKEE-EQIGKCSTRGR	0.93738
2	73–91	18	CAVLSCLPKEE-RGRKCCRRKKE	0.64715
3	83–91	8	EQIGKCSTRGR- RGRKCCRRKKE	0.99884

### Analysis of disulfide engineering

Using the DiANNA tool, three disulfide bonds were identified at the β-defensin-2 epitopes, which act as TLR4 agonists. This suggests that the stability of the construct’s folding is helped by these bonds (Table 11).

An analysis of the vaccine construct for disulfide engineering revealed that there are 4 number of residue pairs that can effectively form disulfide bonds due to their geometric properties. The pair (ALA 9-GLU 186, ALA 13-ALA 19, ALA 114-MET 159 and GLU 273-ALA 324) stood out with optimal conditions, presenting a Chi3 angle of (−85.99, −87.82, −76.18 and − 80.29)°, a Cβ–Cβ distance of (4.4–6.8) Å, and an energy score of (0.37, 0.47, 1.39 and 1.83 kcal/mol), suggesting a strong likelihood of creating a stable disulfide bond. Other viable pairs included. Adding these predicted disulfide bonds is anticipated to improve the vaccine’s thermostability and structural integrity, which may enhance its stability in vivo while keeping key epitopes fully exposed (Table 11).

**Table 11:** Disulfide engineering analysis by disulfide by design (DbD2).

No.	Residue sequence 1	Residue sequence 2	Chi3	Energy	Sum B factor
1	ALA 9	GLU 186	−85.99	0.37	25.12
2	ALA 13	ALA 19	−87.82	0.47	23.36
3	ALA 114	MET 159	−76.18	1.39	25.58
4	GLU 273	ALA 324	−80.29	1.83	24.02

####  Prediction of surface accessible and hydrophilicity regions

**Fig. 7:**
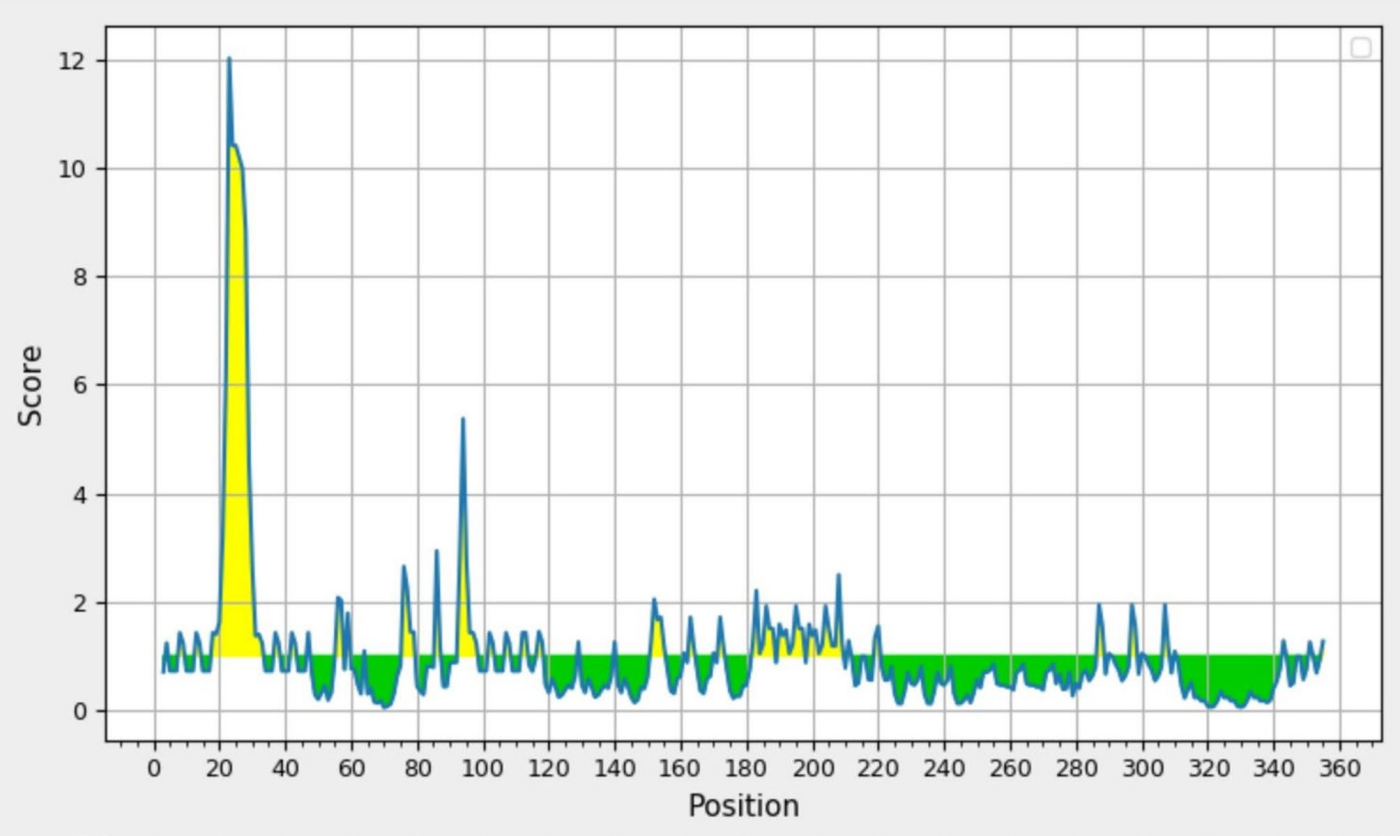
Surface accessibility of Tat and β-defensin peptides. Surface accessible epitopes are yellow regions above the threshold. The x-axis show the amino acid sequence position and y-axis show the surface accessibility score. The threshold was 1.000.

**Fig. 8:**
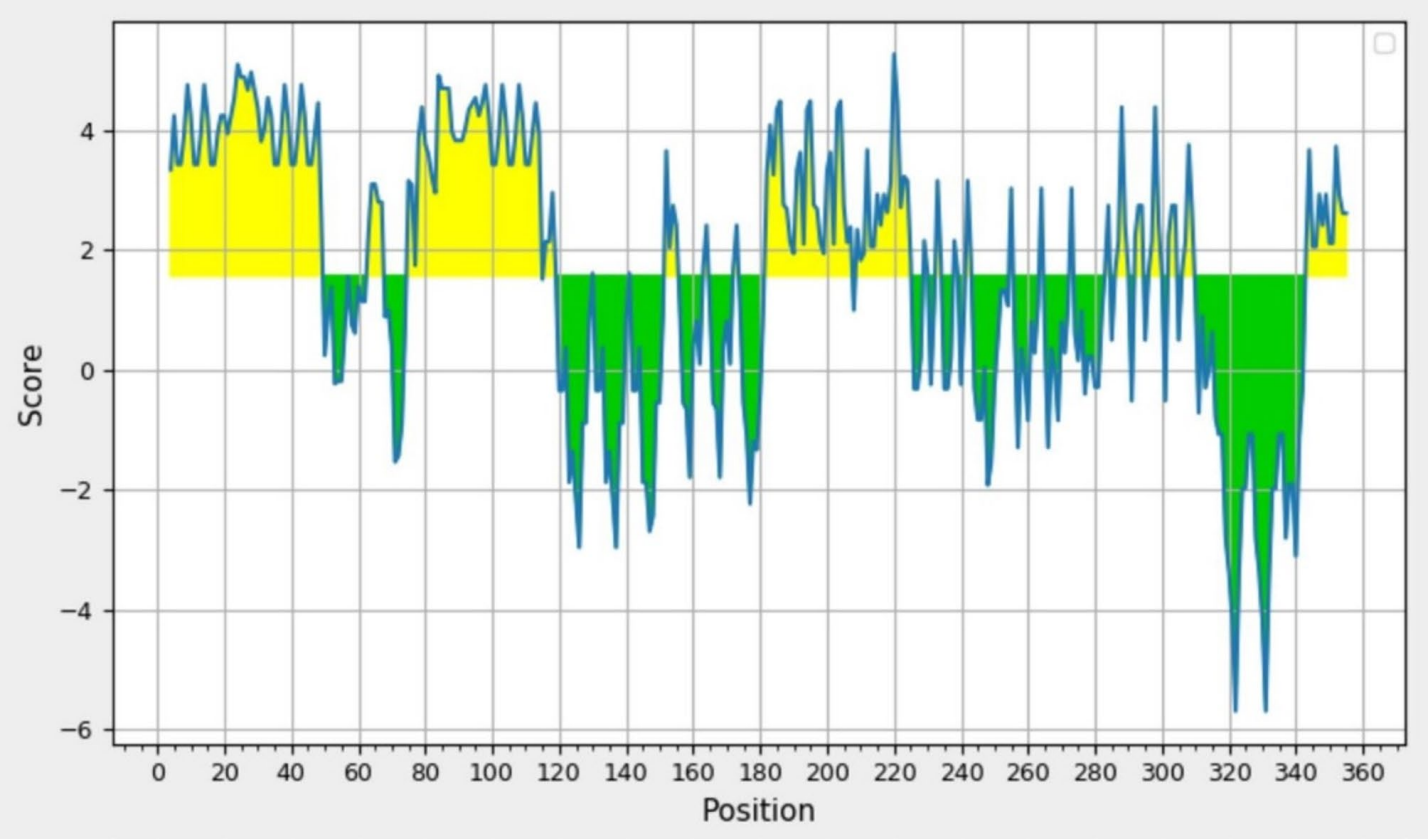
Parker hydrophilicity prediction of the Tat and β-defensin peptides. The x-axis and y-axis show the position and score, respectively. The threshold was 1.569. The regions are hydrophilic in the protein above the threshold value, are shown in yellow color.

Surface accessibility of HIV-1 Tat (as CPPs) and β-defensin (as TLR4 agonist) epitopes is essential because hydrophilic regions are commonly positioned on the surface to deliver drugs into cells and interact with the receptor, respectively. At threshold cutoffs 1.000 and 1.569, the surface accessibility and hydrophilic regions, respectively, of the HIV-1 Tat as a CPP protein and β-defensin as a TLR4 agonist were evaluated by Emini and Parker tools; Tat peptide and β-defensin were found to have scores above the threshold (Figs. 7 and 8, respectively).

### Prediction of secondary structure

**Fig. 9:**
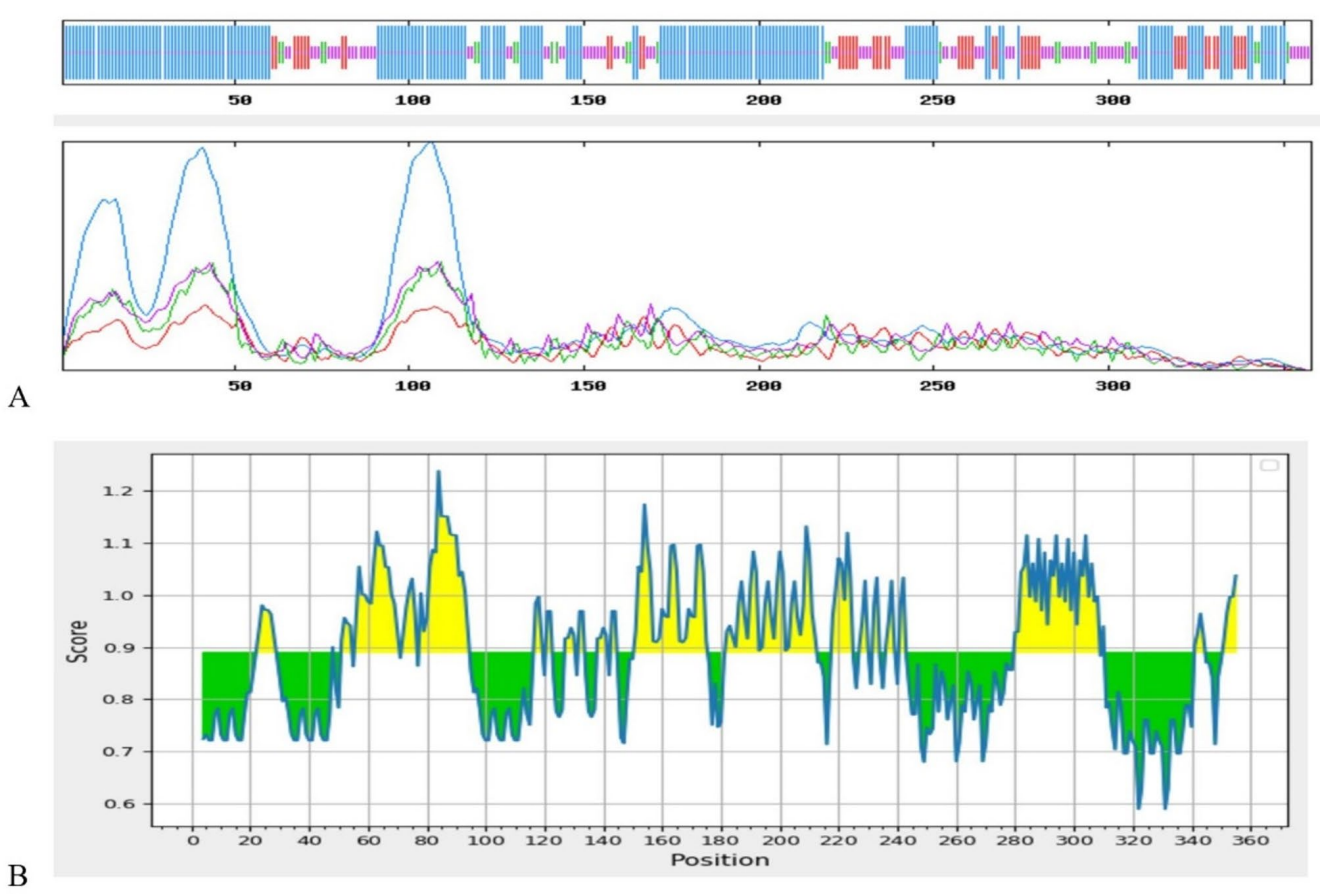
Secondary structure prediction of platform construct. **(A)** Secondary structure prediction of platform construct using the SOMPA server having (55.31%) α-helix, (6.98%) β-strand, (13.69%) extended strand and (24.02)% random coil. **(B)** Chou and Fasman β-turn prediction of the platform peptides specially, β-defensin. The x-axis and y-axis show the position and score, respectively. The threshold was 0.890. The regions are β-turn in the protein above the threshold value, are shown in yellow color.

The overall platform sequence was assessment by using SOPMA tool to have 55.31% α-helix, 6.98% β-strand, 13.69% extended strand and 24.02% random coil (Fig. 9A). Furthermore, Chou and Fasman β-turn prediction that β-defensin (as TLR4 agonist) amino acid residues would be exposed for binding to the receptor (Fig. 9B).

### Tertiary structure modelling and refinement

**Fig. 10:**
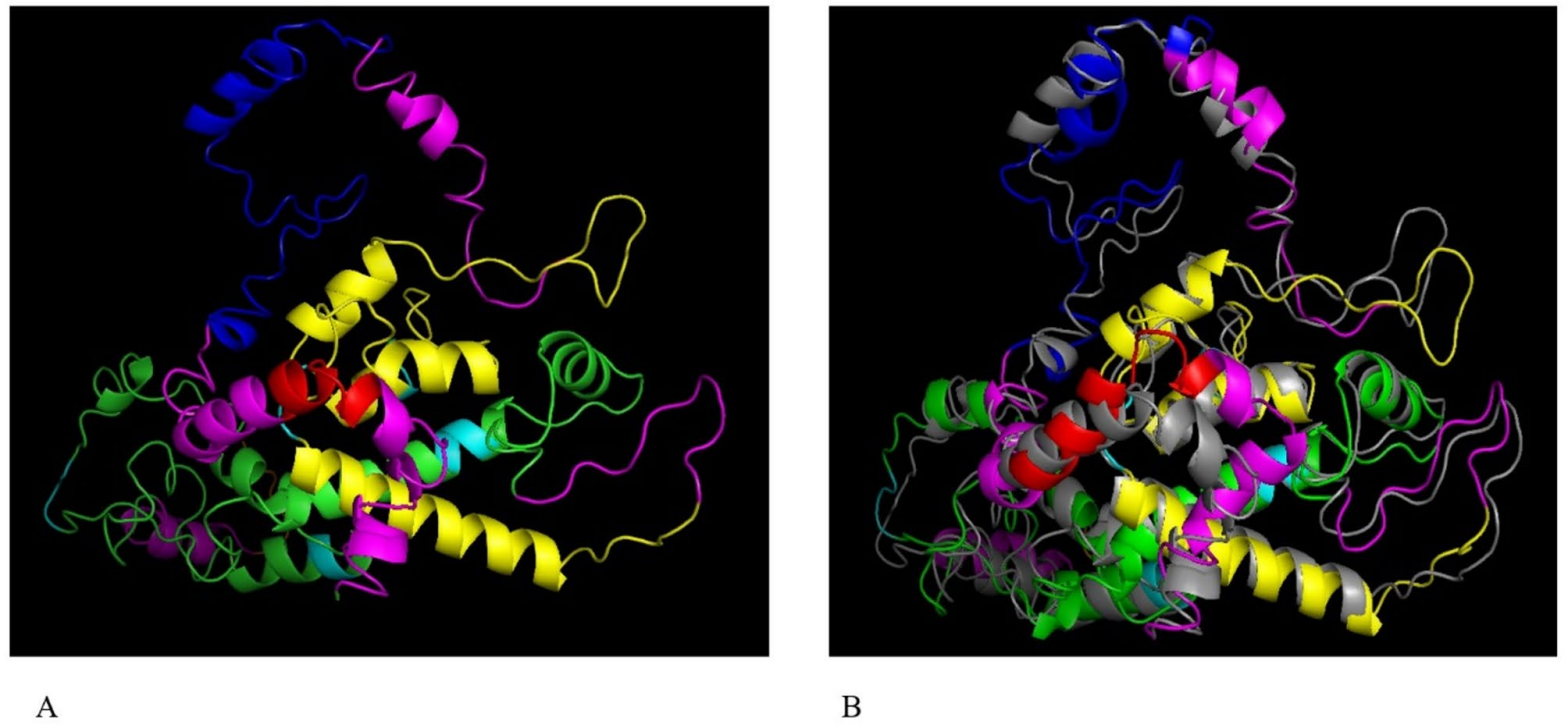
Protein 3D structural modelling and refinement. **(A)** The 3D model of the platform was obtained on the I-TASSER server. **(B)** Alignment by the DeepRefiner server of a refined 3D structure (colored) on a crude model (gray).

The I-TASSER server generated five 3D structural models of the platform, utilizing ten threading templates, with C-scores ranging from − 3.47 to −2.45 and Z-scores from 1.10 to 4.56. Given that C-scores range from − 5 to 2, with higher values indicating greater confidence, the model with the highest C-score of −2.45 was selected for further analysis (Fig. 10A). This model exhibited a TM-score of 0.43 ± 0.14 and an expected root-mean-square deviation (RMSD) of 12.4 ± 4.3 Å. The TM-score, a measure of structural similarity less sensitive to native errors than RMSD, was employed to assess model quality.

The DeepRefiner server was used to enhance the stability and energy minimization of the modeled protein. The refinement of the platform model on the DeepRefiner server presented five model structures. The quality of the structure model 1, based on numerous factors, such as MolProbity (1.741), GDT-HA (60.545), RMSD (2.270) and RW-plus score (−54973.924964), was the most significant. The global quality factor from 0 to 1, with high scores indicating high quality that was 0.110 for model 1 (Fig. 10B). This model was chosen for the validation study.

### Tertiary structure validation

**Fig. 11:**
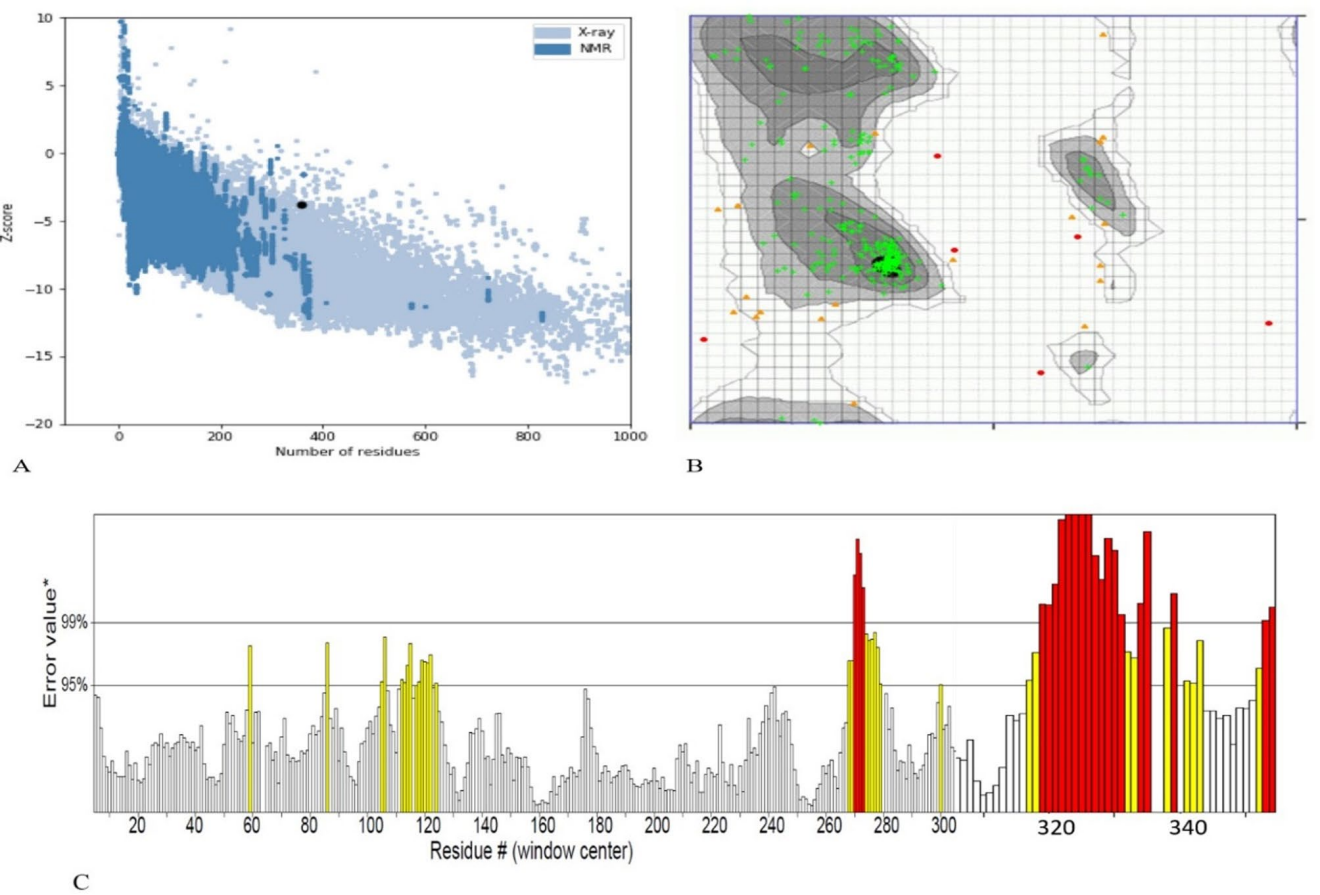
Validation 3D structure of the platform. **(A)** ProSA server with a Z-score of −3.74. **(B)** Ramachandran plot analyses show 91.92% highly preferred, 6.21% preferred and 1.8% questionable regions of protein residues. **(C)** The ERRAT plot analysis showed that the overall quality factor of the refined construct is as high as 84.01%**. * In the error axis, two lines were drawn to ensure that areas that are higher than the error value can be passed. **Expressed as the percentage of the protein. The calculated error value falls below the 95% rejection threshold. High-resolution structures generally produce values of about 95% or higher. For low resolution (2.5 to 3 A), the average overall quality factor is about 91%. All three servers confirmed the quality and possible errors in the 3D model.

Quality assessment of the 3D model, using ProSA and ERRAT servers, demonstrated its validity. ProSA produced a Z-score of −3.74 (Fig. 11A), and ERRAT reported an overall quality factor of 84.12% for the refined model (Fig. 11C). Ramachandran plot analysis revealed a high percentage of residues within favored regions: 91.92% in highly favored, 6.21% in favored, and 1.86% in questionable regions (Fig. 11B). These results, obtained from ProSA, ERRAT, and Ramachandran plot analyses, collectively validated the accuracy of the 3D modeled protein.

### Protein-protein molecular docking

**Fig.12:**
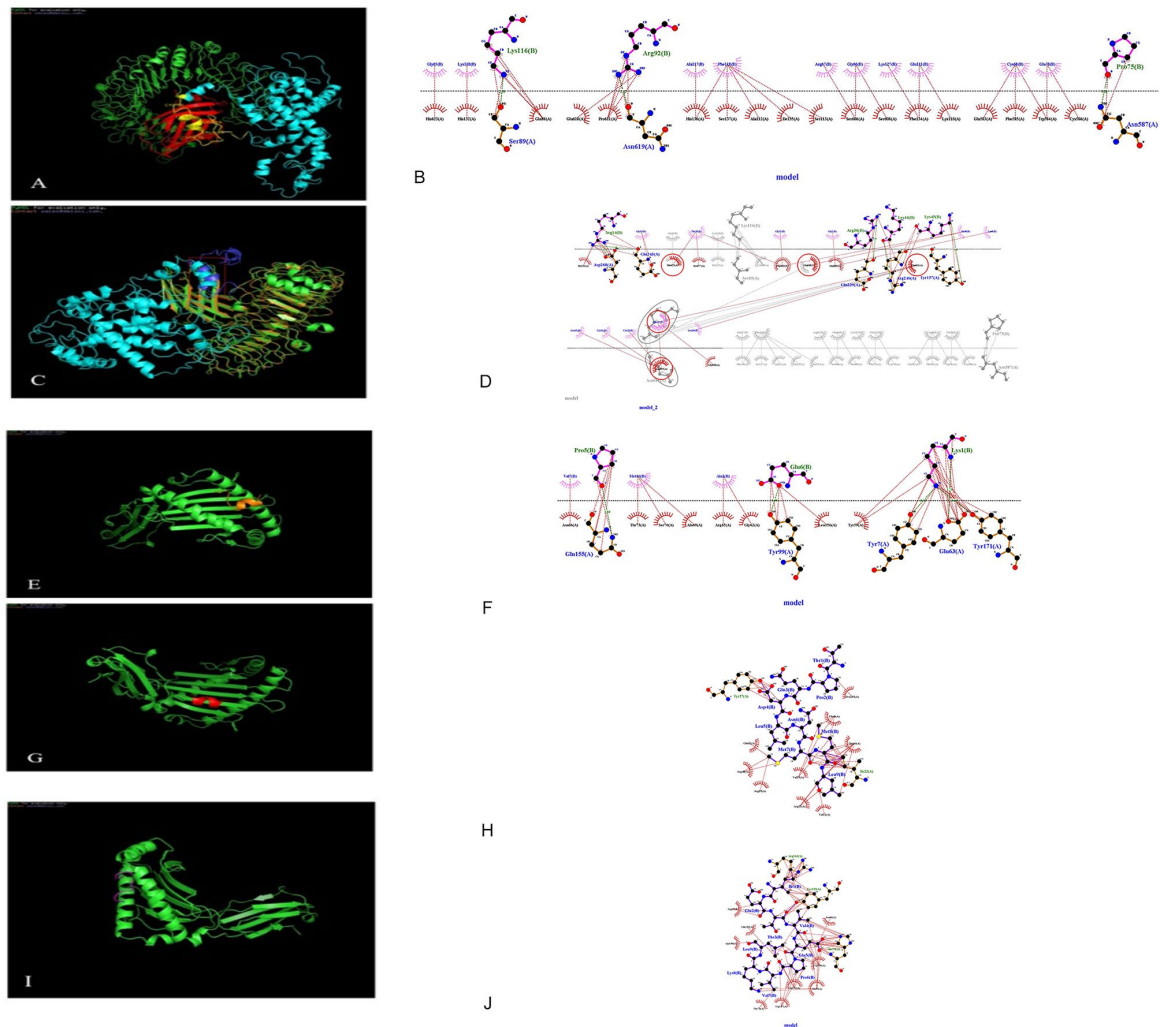
Analyses of the molecular docked complex between platform and MD2/TLR4, CTL epitope and MHC-I and analysis of the hydrogen bonds and hydrophobic interactions (The green lines are hydrogen bonds and the red lines are hydrophobic interactions). **(A)** 3D structure of molecular docking simulation between TLR4/MD2 and the β-defensin epitope in the platform construct. The cyans chain is the platform construct, the yellow ribbon is β-defensin epitope in the construct, the red chain is MD2, and the green chain is TLR4. Results of the interaction analyses of the platform with TLR4 by HawkDock server showed good affinities between the receptor (TLR4) and the β-defensin (TLR4 agonists) modeled platform. **(B)** Hydrophobic interactions by Gly 65,Gly 66, Arg 67, Cys 68, Glu 78 and Arg 92 of β-defensin in platform with MD-2/TLR4 receptor. Results of these analyses indicated the proper interactions of ligand β-defensin within platform to MD2/TLR4 receptor, that might activate NFkB signaling pathway. **(C)** Alignment of molecular docking of natural β-defensin with MD2/TLR4 as a control complex and β-defensin platform construct with MD2/TLR4. The orange chain is MD2/TLR4, the blue chain is natural β-defensin, the green chain is MD2/TLR4 (docked with platform), the cyan is platform. **(D)** Alignment of hydrophobic interactions by Glu27 in natural β-defensin as a control complex which is equivalent to it on the β-defensin platform within platform Glu 78 to MD2/TLR4 receptor. **(E)** 3D structure of molecular docking simulation between HLA-B*57:01 and the KF11 epitope in the platform construct. The orange chain is the KF11 construct, the green chain is HLA-B*57:01. **(F)** The interaction of Lys 1, Pro 5 and Glu 6 of in the KF11 with HLA-B*57:01 is shown. The hydrogen bond lengths are shorter than 2.84 Å. **(G)** 3D structure of molecular docking simulation between HLA-B*07:02 and the TL9 epitope construct. The red chain is the TL9 construct, the green chain is HLA-B*07:02. **(H)** The interaction of Pro 2, Asp 4, Met 7, Met 8 and Leu 9 of in the TL9 with HLA-B*07:02 is shown. **(I)** 3D structure of molecular docking simulation between HLA-A*25:01and the Pol RT 5–12 epitope construct. The pink chain is the Pol RT 5–12 construct, the green chain is HLA-A*25:01. **(J)** The interaction of Ile 1, Glu 2, Thr 3, Val 4, Glu 5, Pro 6, Val 7 and Leu 9 of in the Pol RT 5–12 with HLA-A*25:01 is shown.

Studies to properly design TLR4 modulators consider the LPS binding site as the canonical binding site. However, since most of the ligands are derived from the LPS structure, molecular docking was necessary to confirm the ability of β-defensin to be a therapeutic target because of its different nature^64^. The results analysis of the docking between ligand and receptor indicated a high HowkDock score for β-defensin of the platform interaction (E-total of −4376.95 kcal/mol), demonstrating the good affinity between the MD2/TLR4 receptor. Furthermore, the results of the molecular docking simulated between β-defensin of the platform construct and the MD2/TLR4 could efficiently bind to the receptor (Fig. 12A), which the alignment of the control dock complex with the platform dock complex is shown was similar to the natural counterpart with an E-total of −4818.23 kcol/mol for β-defensin interaction with MD2/TLR4 complex (Fig. 12B). The results showed the proper interactions of MD2/TLR4 receptor with β-defensin in the platform, that was similar to the natural ligand interaction. Finally, the hydrogen bonds and hydrophobic interactions between the platform and MD2/TLR4 (Fig. 12C) was evaluated by Dimplot analysis.

We identified three CTL epitopes with high affinity, chosen based on predictions from IEDB regarding binding and antigenicity: KF11 (KAFSPEVIPMF), TL9 (TPQDLNMML), and Pol RT (IETVEPVKL). The structures of the peptides were created using PEP-FOLD3. For the molecular docking between epitopes and MHC-I, we utilized HowkDock. The binding poses were selected according to energy scores (KF11; −2512.90, TL9; −2268.10 and Pol RT; −2641.63) (Fig. 12D, F, H). All three epitopes showed stable binding in the peptide-binding cleft of MHC-I, with E-total (KF11; −24.59 kcal/mol, TL9; −17.34 kcal/mol and Pol RT; −25.17 kcal/mol). There were hydrogen bonds and hydrophobic interactions with important anchor residues of MHC KF11 (KAFSPEVIPMF) with HLA-B*57:01, TL9 (TPQDLNMML) with HLA-B*07:02 and Pol RT 5–12 (IETVEPVKL) with HLA-A*25:01, indicating predicted high affinity and correct positioning for T-cell receptor recognition (Fig. 12E, G, I).

### Molecular dynamic simulation

**Fig. 13:**
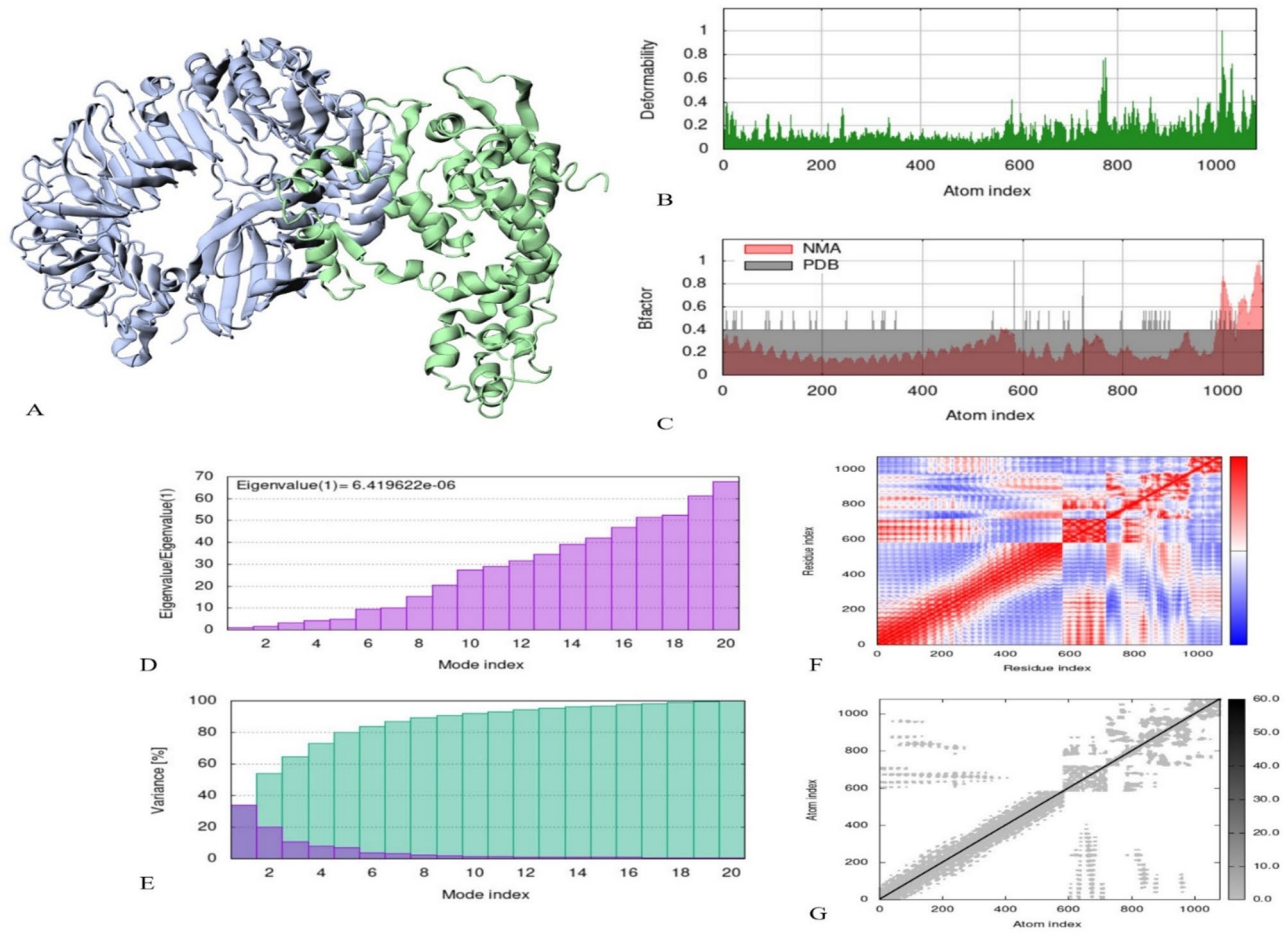
Analyses of the molecular dynamics simulation of platform and TLR4 docked complex. **(A)** 3D structure of NMA mobility. The purple chain is MD2/TLR4, and the green chain is platform. **(B)** Deformability plot, the peaks in the graph are deformability regions of the protein. **(C)** B-factor diagram. **(D)** Eigenvalues graph. **(E)** Variance graph, red color is individual and green color is cumulative variances. **(F)** Co-variance diagram, red, white and blue color are correlated, uncorrelated and anti-correlated, respectively. **(G)** Elastic network plot, darker gray color area are stiffer regions.

The dynamic behavior of the platform construct and its docked complex with TLR4 was assessed through molecular dynamics simulation and normal mode analysis (NMA) (Fig. 13A). The B-factor/mobility simulation was done to conclude the mobility of atoms and molecules in the platform construct. Deformability plots revealed regions of flexibility within the platform-MD2/TLR4 complex (Fig. 13B). The B-factor diagram provides a clear picture of the docked complex relationship between NMA and the PDB fragment (Fig. 13C). The docked complex eigenvalue was determined to be 6.419622e-06 (Fig. 13D), with variance inversely related to eigenvalue (cumulative, green; individual, red) (Fig. 13E). Residue covariance within the docked complex was shown, indicating correlated (red), uncorrelated (white), and anti-correlated (blue) movements (Fig. 13F). Elastic network modeling illustrated atomic interactions, with darker gray regions representing areas of increased rigidity (Fig. 13G).

### Immune simulation

**Fig. 14:**
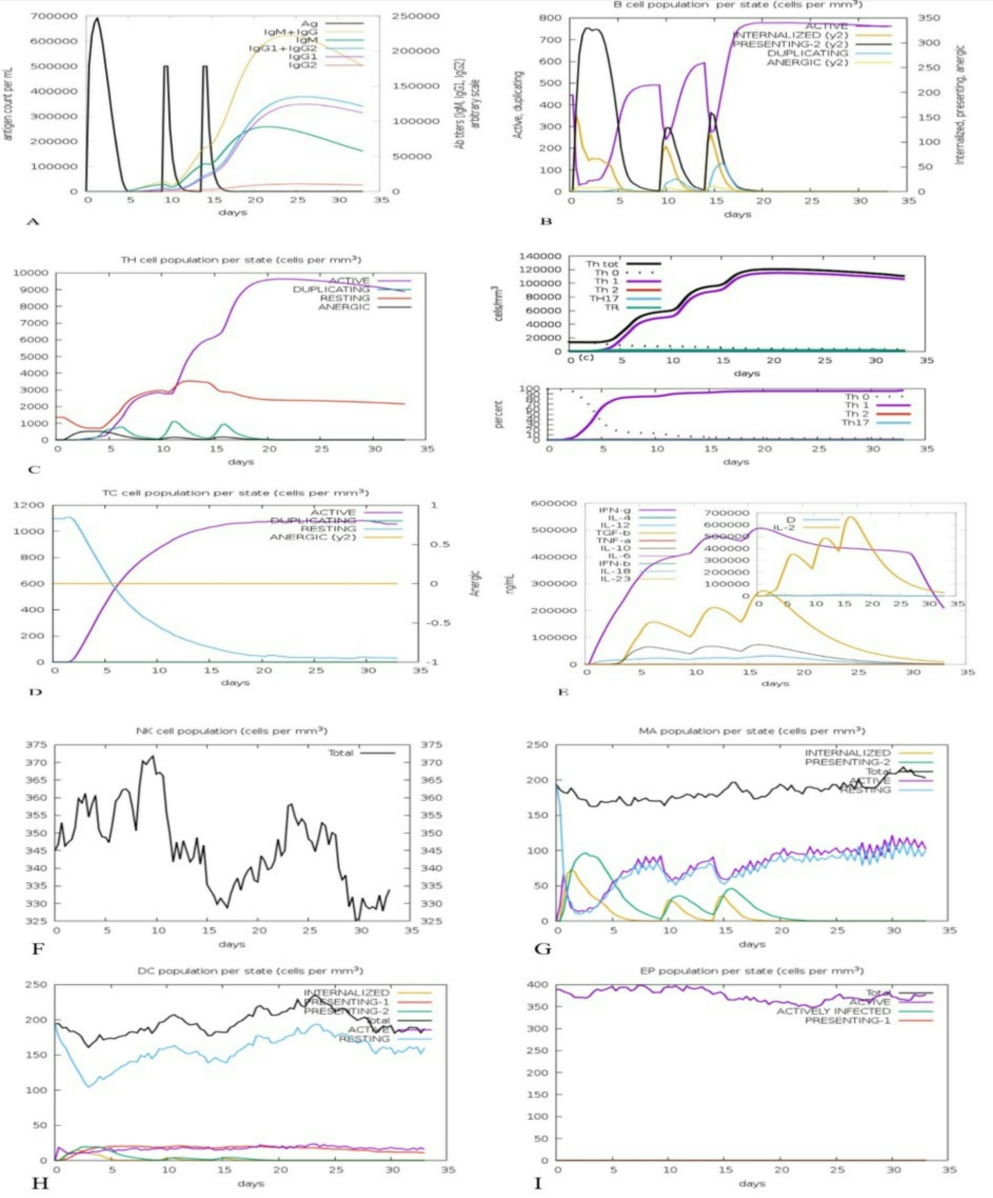
In silico immune simulation analyses of the vaccine design (platform) by C-ImmSim. **(A)** Production of immunoglobulins titers after injection and response to platform (black vertical lines). **(B)** B cell population per state prediction after the injections that show B cell is active. **(C)** The Th cell population per state levels and percent after the injection and show Th1 was induced to about 100%. **(D)** The T cytotoxic population per state after the injections that show TC is active. **(E)** The plot shows interleukins and cytokines levels after the injections. Another plot shows IL-2 level with the Simpson index and D shows by the blue line. An increase in D line over time show the emergence of epitope dominant clones of T cells and the smaller the value of D, the lower the variety. High titers of IFN-γ and TGF-b were induced after injections. **(F)** Total number of natural killer cells. **(G)** Total number of active, resting, Ag internalized, and Ag presenting dendritic cells. **(H)** Total count, Ag internalized, active and resting macrophages. **(I)** Total count of epithelial cells broken down to active.

C-ImmSim studies sequential and effective innate and adaptive immune responses, cell state and immune cell memory. The simulation showed a strong primary and secondary antibody reaction. Notably, after the first injection, a significant level of IgM and IgG antibodies was detected, peaking around day 10. Following additional doses, increased IgG1 and IgG2 levels were seen, indicating a shift in antibody type and the development of immune memory. The observed titers for IgM, IgG1 + IgG2, and total IgM + IgG reached nearly 90,000, 140,000, and 230,000, respectively, while antigen levels decreased, signifying effective removal of the antigen (Fig. 14A, B). A marked increase in CD4 + helper T cells, especially of the Th1 type, was recorded after the second and third vaccinations. The population of CD8 + cytotoxic T lymphocytes (CTLs) grew significantly, with many cells becoming actively engaged, aligning with the CTL-epitope-based design. Both CD4 + and CD8 + T cell populations showed a formation of lasting memory cells, which enhances long-term cellular immunity (Fig. 14C, D). Following immunization, high quantities of IFN-γ and TGF-β were generated, pointing to a Th1-dominated response that supports antiviral immunity. The persistent rise of these cytokines throughout the simulation underscores the platform’s capability to promote cellular immune clearance processes (Fig. 14E). The vaccine formulation successfully activated dendritic cells (DCs), macrophages, and NK cells early on, sustaining their presence during the simulation. These innate immune reactions further reinforce the potential for effective antigen presentation and T-cell activation (Fig. 14F, G, and H). For 30 days, epithelial cells remained actively responsive, showing that the vaccine does not cause exhaustion or inhibit tissue barrier functions (Fig. 14I).

### Expression and purification of recombinant protein

**Fig. 15:**
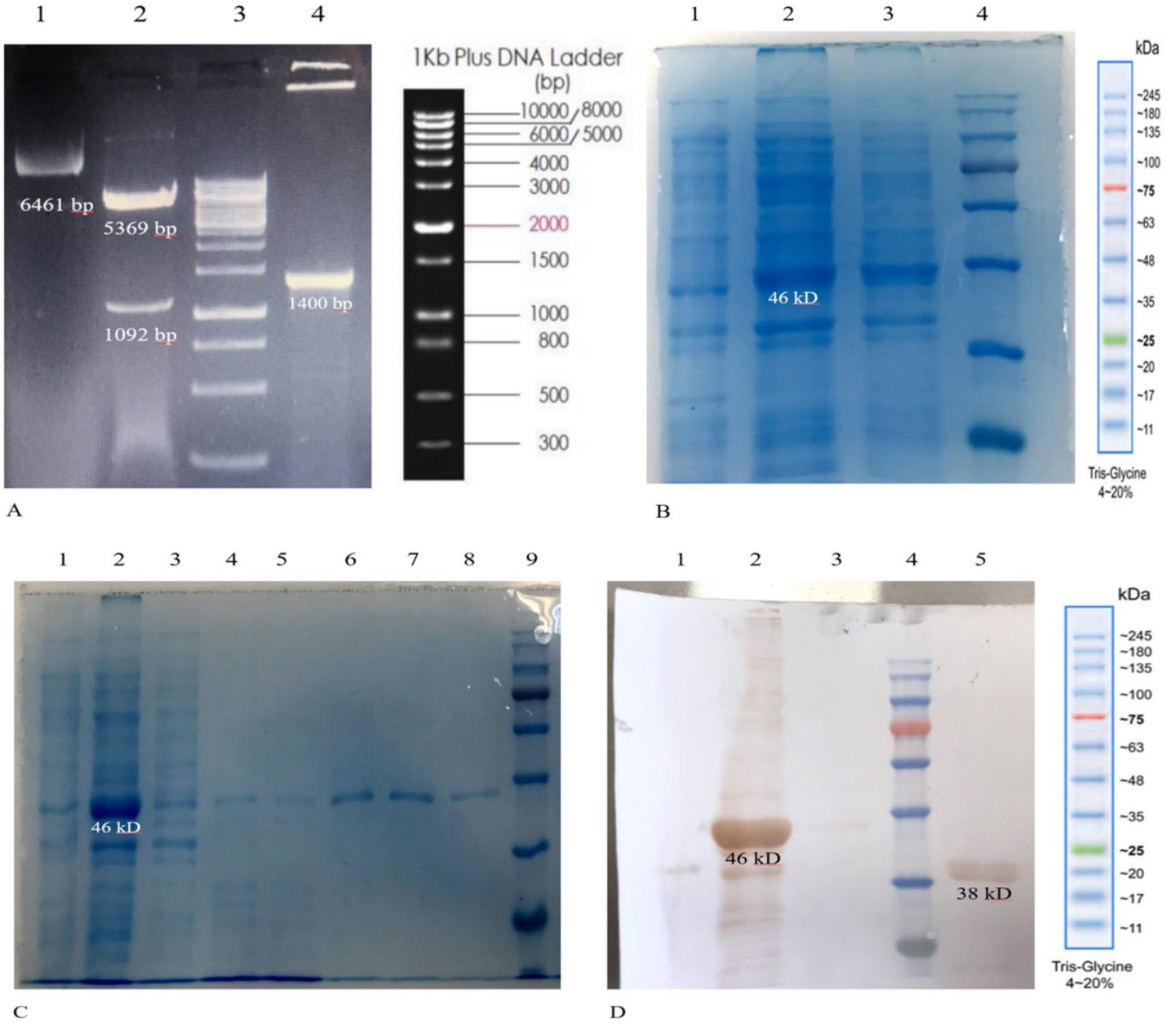
Restriction analysis of the pET28a vector encoded DNA and characterization of the *E. coli*-derived RP protein by SDS-PAGE and WB analyses. **(A)** Lane 1: Undigested pET28a vector harboring 1092 bp encoded DNA. Lane 2: Digestion with *BamHI* and *Xho*I enzymes resulted in two fragments of 5369 bp and 1092 bp. Lane 3: DNA marker DM3100 (SMOBIO 1 kb, Taiwan). Lane 4: PCR product 1263 bp. **(B)** SDS-PAGE analysis before and after induction of pET28a-RP-harboring *E. coli* with IPTG revealed a protein band of approximately 46 kDa corresponding to the expressed RP. Lane 1: Un-induced cell lysates of the *E. coli* BL-21(DE3) cells harboring pET28a-RP. Lane 2: Cell lysates of *E.coli* cells harboring pET28a-RP after 4 h induction with IPTG. Lane 3: Cell lysates of *E.coli* cells harboring pET28a-RP after 16 h induction with IPTG. Lane 4: Molecular weight marker (Sinaclon SL7002, Iran). **(C)** SDS-PAGE analysis native purification of pET28a-RP-harboring *E. coli.* Lane 1: Un-induced cell lysates of the *E. coli* BL-21(DE3) cells harboring pET28a-RP. Lane 2: Crude lysates of *E.coli* cells harboring pET28a-RP before centrifuge. Lane 3: Flow throw. Lane 4: Washed (1) Lane 5: Washed (2) Lane 6: Elution 1 of RP. Lane 7: Elution 2 of RP. Lane 8: Elution 3 of RP. Lane 9: Molecular weight marker (Sinaclon SL7002, Iran). **D)** Western blotting results for the expressed RP. Lane 1: Un-induced cell lysates of the *E. coli* BL-21(DE3) cells harboring pET28a-RP. Lane 2: Crude lysates of *E.coli* cells harboring pET28a-RP after 4 h induction with IPTG. Lane 3: Elution 1 of RP. Lane 4: Molecular weight marker (Sinaclon SL7002, Iran). Lane 5: HPV L2 protein as a positive control with 38 kD as MW.

The pET28a vector containing the 1092 bp platform-encoding DNA sequence was confirmed via restriction analysis using *BamHI* and *XhoI* enzyme pairs (Fig. 15A). IPTG induction of *E. coli* BL21 (DE3) cells harboring the pET28a-platform construct resulted in the expression of a recombinant protein with an approximate molecular weight of 46 kDa (Fig. 15B). Purification of the recombinant protein using Ni-NTA chromatography under native conditions yielded a homogenous protein band (Fig. 15C), with an approximate yield of 0.4 mg/ml. Western blot analysis further confirmed the identity of the recombinant protein (Fig. 15D). Endotoxin levels in the purified platform were determined to be below 0.01 EU/ml, meeting the requirements for immunization studies.

## Discussion

As interest in T cell therapies for HIV grows, it is crucial to carefully examine the specificity of CTL responses. Research on HIV controllers shows that these individuals often have an abundance of certain MHCI alleles, which likely results from CTL activity against viral regions that are resistant to mutations^19^. Therefore, combining these MHCI alleles with specific CTL epitopes becomes a vital approach for managing HIV latency. To effectively control latency, creating therapeutic vaccines based on these insights is necessary. Utilizing advanced bioinformatics tools can improve vaccine design more than traditional methods^65,66^. Identification of immunogenic antigens is essential in vaccine design because they have a unique property to bind and respond to immune cells^67^. To achieve these findings, we first designed a platform including the HIV-Tat (as a CPP) and β-defenisin-2 (the TLR4 agonist) fused to the 3x tandem repeats of the CTL epitopes Gag (KF11, TL9 and QW9) and Pol (PR; 68–76 aa, RT; 5–12 aa, 117–126 aa, 375–383 aa) by short rigid linkers of (EAAAK)_4_ and HEYGAEALERAG (Fig. 2).

CTL epitopes are essential for the stimulation of adaptive immunity and the control of HIV-1 latency and are required for cooperation with MHC class I and class II molecules^17,19^. For developing multi-epitope-based vaccines, recognition of CD4^+^ and CD8^+^ T cells is necessary^68^. We used bioinformatic tools to conduct a reliable investigation to evaluate the platform containing two Gag p24 and Pol region epitopes that make up HIV-1 therapeutic vaccines. The results of analyzing the binding of the epitopes to mouse MHCI alleles revealed that the 9-mer IETVEPVKL, TPQDLNMML and KAFSPEVIPMF epitopes form Pol (RT; 5–12 aa), Gag (TL9) and Gag (KF11) were suitable CTL epitopes in mouse (Table 1). The results of analyzing the binding of the epitopes to human MHCI alleles showed that the 9-mer IAMESIVIW, IETVEPVKL, QASQEVKNW and TPQDLNMML epitopes form Pol (RT; 375–383 aa), Pol (RT; 5–12 aa), Gag (QW9) and Gag (TL9) were the suitable CTL epitopes in human (Table 2). Interestingly, our results demonstrated that the three repeats of Pol (RT; 375–383 aa) in mice (Table 3), Pol (RT; 375–383 aa) and Gag (KF11) epitopes in humans interact with MHCI and II molecules (Table 4). These results, in agreement with the previous studies, indicated that CTL epitopes play a vital role in the treatment of HIV latency^69,70^. Combine MHCI and II alleles, for which platform containing CTL epitopes showed affinity, were searched for population coverage. The highest population coverage infected with HIV-1 was recorded in Europe with 81.87%, and after Europe were 78.51%, 73.57% and 71.29% coverage from Iran, North America, and the West Indies, respectively (Fig. 4; Table 7). Our vaccine development focuses on CTL epitopes to combat latent HIV-1 infections, and we have implemented various strategies to tackle common drawbacks found in CTL-based vaccines. Initially, we did not directly include linear B-cell epitopes; however, we used the ElliPro server to anticipate conformation-based (discontinuous) B-cell epitopes (Fig. 3; Table 6) from the 3D model of our construct. Notably, these epitopes in the β-defensin and Tat regions are accessible on the surface and hydrophilic, suggesting they could trigger humoral immune responses that are crucial for neutralizing viruses present outside cells. Furthermore, to address the challenges posed by MHC class I limitations and HLA diversity, we selected various CTL epitopes from conserved sections of Gag and Pol proteins (such as KF11, TL9, IETVEPVKL, IAMESIVIW) known for their strong immune response and ability to resist mutations. These epitopes are also expected to bind effectively to a wide range of human HLA types, and our analysis of population coverage using the IEDB tool indicated extensive global representation, with over 78% in Iran, 81% in Europe, and significant coverage in North America and Asia. Finally, by incorporating multiple highly conserved and functionally critical epitopes, we minimize the chance of immune evasion. Hence, while CTL responses are naturally limited by MHC-I, our design has been carefully crafted to enhance coverage, stability, and immune activation potential, potentially facilitating both cellular and humoral responses through the predicted B-cell epitope areas.

The non-allergenicity and safety of the designed peptide vaccine have increased its efficiency as a candidate vaccine (Table 8). Furthermore, the designed peptide-based vaccine showed a good antigen score in the Vaxijen v2.0 server (Table 8). and CTL epitopes as antigens in Kolaskar and Tongaonkar tool (Fig. 5). Immunoinformatic analyses indicated the immunogenicity and safety of the platform as an immunogenic peptide.

As shown in Table 10; Fig. 6 results of physicochemical properties analysis showed that the platform with 359 amino acids and 46 kDa molecular weight is a stable protein (94%) with high expression in *E. coli*. The expected theoretical pI is 9.25, signifying that the platform construct is alkaline. Predicted high stability with an instability index of 38.41 indicated that the protein will be very stable after expression. There are aliphatic side chains in the protein, which indicates its hydrophobicity. On the other hand, the negative GRAVY index in the protein signifies that the protein is hydrophilic. The higher aliphatic index of 81.45 for the designed platform with suitable GRAVY of −0.266 showed the platform is hydrophilic (Fig. 8) that is in accordance with the percentage of α-helix and random coiled in the secondary structure (Fig. 9A). These results, in agreement with previous studies, demonstrated the importance the percentage of α-helix and coiled areas in the secondary structure of the designed construct immunogen for proper solubility and stability^71^.

Figures 7 and 8 illustrate that for effective drug delivery into cells, the accessibility of the Tat epitope on the surface is essential, as hydrophilic areas typically play a role in this exposure^26^. Additionally, it is important for β-defensin, functioning as a TLR4 agonist, to be present on the surface to facilitate interactions with MD2/TLR4^72^. Analyses from prediction tools for Emini surface accessibility and Parker hydrophilicity indicated that the segments spanning 22–30 and 51–95 are both accessible and hydrophilic, relating to the epitopes of Tat and β-defensin (the TLR4 agonist).

When synthetic peptides are examined, two types of secondary and tertiary structures can be formed in their native structure that are detected in response to infection^73^. The quality of the 3D structure of the designed vaccine enhanced significantly following refinement (Fig. 10) and displayed the appropriate amino acid properties according to the results of the ProSA, ERRAT, and Ramachandran plots (Fig. 11). The result of the Ramachandran plot demonstrates that 91.92% of residues in the highly preferred regions, 6.21% of residues in the preferred regions and 1.86% in the questionable regions, demonstrating the acceptable quality of the 3D model. Aftab et al.^74^ created a combined model that uses deep learning and ordinary differential equation (ODE) integration to predict how vaccine-like peptides pass through the blood-brain barrier. This research shows the benefits of merging neural networks with mechanistic models. We mention this to back our approach of using I-TASSER along with MD simulation, which strengthens the reliable methods for predicting structure and function in the design of immunogens.

In our analysis of the structure, we used the DiANNA server to predict disulfide bonds, which revealed three natural disulfide bridges in the β-defensin-2 area of the construct. These bonds play a crucial role in preserving the native shape and functional effectiveness of β-defensin as a TLR4 agonist. Through the DbD2 analysis of disulfide engineering, three residue pairs were found to be significant (ALA 9-GLU 186, ALA 13-ALA 19, ALA 114-MET 159 and GLU 273-ALA 324). These pairs showed good Chi3 angles, ideal Cβ distances, and low energy scores, suggesting that these modified bonds could enhance the vaccine’s structural stability while still allowing for easy access to epitopes.

To investigate the ability of the vaccine designed (β-defensin) to interact with TLR4 on immune cells, TLR4 was docked to the vaccine designed (Fig. 12). The results showed that the vaccine designed (β-defensin) had a high interacting and binding affinity towards MD2/TLR4, which has been previously reported for the LPS and TLR4^75^. This interface of vaccine (β-defensin) with TLR4 signifies that vaccine candidates have the potential to induce both innate and adaptive immune responses. In this study, we conducted molecular docking involving the vaccine construct we designed and the murine TLR4/MD2 complex (PDB ID: 3VQ2). Although our target vaccine is for humans, we opted for the mouse TLR4 structure because a complete, high-resolution crystal structure of the human TLR4/MD2 complex with peptide ligands, particularly β-defensin, is unavailable. It is noteworthy that mouse TLR4 and human TLR4 have a significant structural and functional similarity, especially in their ligand-binding areas, and murine TLR4 is often regarded as a reliable model for studying TLR4-ligand interactions. Additionally, murine models are frequently employed in preclinical immunization studies, making findings from mouse TLR4 docking quite relevant for evaluating the vaccine’s potential effectiveness in these contexts. Moving forward, we aim to carry out homology modeling or take advantage of newly available structural data for human TLR4 to validate these interactions and emphasize their significance for translation.

Qin et al.^76^ used deep learning to choose scaffolds and conducted extensive tests to find small-molecule receptor activators. Although they targeted a different receptor (Adenosine A₂B), their method is similar to our comprehensive approach. We combine binding predictions, structural modeling, and docking to select epitopes and engage TLR4.

This research primarily aimed to create a vaccine that not only stimulates strong CTL responses but also activates hidden HIV-1 reservoirs by focusing on the innate immune system. We included β-defensin-2, recognized as a TLR4 agonist, to specifically activate the TLR4-mediated NF-κB signaling pathway, which is essential for the transcriptional activation of dormant proviruses in resting CD4⁺ T cells. By activating this pathway, we can potentially reverse latency, leading to the exposure of infected cells for immune response. Therefore, our molecular docking studies were centered on assessing how the vaccine construct interacts with the TLR4/MD2 complex. While demonstrating the docking of individual CTL epitopes with MHC class I molecules could provide structural support for epitope presentation, extensive in silico predictions regarding MHC binding had already been conducted using IEDB tools, which are recognized for their high-throughput screening capabilities. Since the goal is to reverse latency through the activation of the innate immune system, we prioritized the interactions with TLR4 and NF-κB activation rather than the structural support of MHC-peptide complexes.

To confirm the accuracy of the predicted CTL epitopes, going beyond just sequence-based binding affinity scores, we conducted molecular docking experiments. This involved interacting chosen epitopes with their associated HLA class I molecules. By using this structure-focused method, we assessed how well the epitopes bind, their stability, and the energy of their interactions within the MHC-I peptide-binding groove. The results from the docking showed that KF11, TL9 and Pol RT epitopes had strong and stable connections with alleles like HLA-A25:01, HLA-B07:02, and HLA-B57:01, which are widely present in populations around the world. Significant interactions, such as hydrogen bonds and hydrophobic connections with key MHC anchor residues, were noted, reinforcing the compatibility and potential immunogenic strength of these chosen epitopes. These results support the trustworthiness of our epitope selection and offer further proof that the vaccine design can effectively engage MHC-I molecules, which is essential for activating CTLs to help eliminate HIV-infected cells.

Molecular dynamic (MD) simulation was performed, investigate the stability and dynamic performance of the β-defensin-MD2/TLR4 complex. The RMSD plot shows the steady binding of the complex (Fig. 13). iMODs server analyzes molecular motion in addition to conformational flexibility by NMA, which is linked by complex coordinates^77^. The NMA of proteins is functionally important because the normal vibrational modes represent minimum frequencies that determine the maximum motions in a protein. The NMA analysis of the docked complex indicated motion and the structural flexibility of the platform (Fig. 13A). Furthermore, our results showed deformability in the platform protein and different peaks with a deformability index of about 1.0 (Fig. 13B). As previously reported, factor B is related to protein mobility, and deformability is a factor of the flexibility of a protein^78^. The B-factor analysis of the platform generated a hinge and average RMS in the B-factor (Fig. 13C). To demonstrate the stiffness of the motion, the set of eigenvalues generated for the docked protein, which correlates with the energy to deform the structure, is examined. The results of the eigenvalues of the docked protein complex showed that it had an amount of deformability (Fig. 13D) which shows a good stability of the molecular motility of the docked protein complexes. The variance graph of the platform complex yielded a reasonable result (Fig. 13E). The co-variance diagram for the platform docked complex showed relatively suitable correlations with few anti-correlations (Fig. 13F). The elastic network plot of the platform complex yielded a reasonable result (Fig. 13G). In this research, an analysis of the vaccine–TLR4 complex was conducted through molecular dynamics by utilizing the iMODS server. This tool employs normal mode analysis (NMA), which evaluates the structural flexibility, stability, and motion of the complex. Although iMODS does not deliver traditional time-dependent molecular dynamics metrics like RMSD, RMSF, radius of gyration (Rg), or hydrogen bond profiles, it serves as an efficient computational alternative that offers important insights into global deformability, eigenvalue-based stability, and correlations between residues. These aspects are especially valuable in the early phases of computational vaccine development when swift evaluation of protein–protein complexes is necessary. The analysis revealed a low eigenvalue for the TLR4–vaccine complex, indicating that only a small amount of energy is needed for conformational changes, which points to favorable binding and structural compatibility. While this initial in silico approach using iMODS was adequate for assessing the overall stability, we recognize the significance of detailed MD simulations for understanding time-dependent dynamic behavior. Therefore, we aim to carry out extensive all-atom MD simulations, such as those with GROMACS, in subsequent studies to explore interaction stability, atomic fluctuations, and the effects of solvents more thoroughly.

The results of the immune simulation showed increased immune responses, which were due to repeated exposure to the antigen. After encountering the antigen, the population of B cells increased, which included a significant amount of antibody titers, respectively, IgM + IgG by 230,000 titers, IgG1 + IgG2 by 140,000 titers, and IgM by 90,000 titers (Fig. 14A, B). The increase in the memory of B and T cells was significantly evident for a long time, especially Th1 was stimulated 100% (Fig. 14C, D). Other notable results were that the levels of IFN-γ and TGF-b increased after the first injection and remained at high levels with repeated antigen exposure (Fig. 14E). The simulation results showed that once the construction began, there was an increase in the quantity of professional antigen-presenting cells (APCs), including NK cells, DCs, and macrophages (Fig. 14F, G, and H). In addition, the epithelial cells remained active from day one all the way to day 30 (Fig. 14I). This indicates the appropriate amount of Th cells and thus the effective production of Ig and cytokines, which contribute to the humoral and cellular immune responses. In several research papers published scientists have effectively explored how peptides interact with receptors through docking and dynamics, especially in immune system contexts. This work strengthens our emphasis on docking the TLR4/MD2 multi-epitope construct, which is essential for validating the mechanisms behind NF-κB activation and reversing latency^79,80^.

We engineered an expression construct for the overproduction of the recombinant protein, in *E. coli* BL21 (DE3). The pET expression vector is a suitable one for high-performance expression of heterologous genes under the control of the T7 lac promoter in *E. coli*^81^. Expression of the 1092 bp DNA sequence corresponding to platform in *E. coli* (BL21) produced a 46 kDa protein (Fig. 15B) that was efficiently purified in the native condition (Fig. 15C). Therefore, our study showed an efficient approach to obtaining recombinant protein with a significantly better purity, which will lower the cost of production and time.

## Conclusion

The bioinformatic methods discussed in this research provide valuable insights into the structure of CTL epitopes, which are essential for therapeutic vaccines against HIV that effectively manage HIV-1 latency. We utilized bioinformatic tools to identify a novel epitope-based vaccine composed of the Gag p24 and Pol proteins fused with Tat and β-defensin into a newly designed construct. This construct shows strong interactions between MHC and CTL epitopes, which aids in triggering a cellular immune response. The platform demonstrates favorable properties as a safe and effective immunogen for a therapeutic vaccine. Molecular docking and MD simulation analyses confirmed that the ligand/receptor complex interacts correctly with significant affinity and stability. Additionally, immune simulations indicated that the vaccine developed can generate a cellular immune response, highlighting that the recombinant protein derived from this method could play a crucial role in controlling HIV-1 latency.

## Data Availability

The authors confirm that all data generated or analysed during the study are included in the submitted manuscript and its information files. The raw data and data that support the findings of this study are available from the corresponding author upon request. There are no restrictions on data availability.
